# Multimodal LLM vs. Human-Measured Features for AI Predictions of Autism in Home Videos

**DOI:** 10.3390/a18110687

**Published:** 2025-10-29

**Authors:** Parnian Azizian, Mohammadmahdi Honarmand, Aditi Jaiswal, Aaron Kline, Kaitlyn Dunlap, Peter Washington, Dennis P. Wall

**Affiliations:** 1 Department of Mechanical Engineering, Stanford University, Stanford, CA 94305, USA; 2 Department of Information and Computer Sciences, University of Hawaii at Manoa, Honolulu, HI 96822, USA; 3 Department of Biomedical Data Science, Stanford University School of Medicine, Stanford, CA 94305, USA; 4 Department of Pediatrics (Clinical Informatics), Stanford University School of Medicine, Stanford, CA 94305, USA; 5 Department of Medicine (Clinical Informatics and Digital Transformation), University of California San Francisco, San Francisco, CA 94143, USA; 6 Department of Psychiatry and Behavioral Sciences, Stanford University School of Medicine, Stanford, CA 94305, USA

**Keywords:** multimodal large language models, autism spectrum disorder, video-based screening, machine learning, artificial intelligence, human-in-the-loop AI

## Abstract

Autism diagnosis remains a critical healthcare challenge, with current assessments contributing to average diagnostic ages of 5 and extending to 8 in underserved populations. With the FDA approval of CanvasDx in 2021, the paradigm of human-in-the-loop AI diagnostics entered the pediatric market as the first medical device for clinically precise autism diagnosis at scale, while fully automated deep learning approaches have remained underdeveloped. However, the importance of early autism detection, ideally before 3 years of age, underscores the value of developing even more automated AI approaches, due to their potentials for scale, reach, and privacy. We present the first systematic evaluation of multimodal LLMs as direct replacements for human annotation in AI-based autism detection. Evaluating seven Gemini model variants (1.5–2.5 series) on 50 YouTube videos shows clear generational progression: version 1.5 models achieve 72–80% accuracy, version 2.0 models reach 80%, and version 2.5 models attain 85–90%, with the best model (2.5 Pro) achieving 89.6% classification accuracy using validated autism detection AI models (LR5)—comparable to the 88% clinical baseline and approaching crowdworker performance of 92–98%. The 24% improvement across two generations suggests the gap is closing. LLMs demonstrate high within-model consistency versus moderate human agreement, with distinct assessment strategies: LLMs focus on language/behavioral markers, crowdworkers prioritize social-emotional engagement, clinicians balance both. While LLMs have yet to match the highest-performing subset of human annotators in their ability to extract behavioral features that are useful for human-in-the-loop AI diagnosis, their rapid improvement and advantages in consistency, scalability, cost, and privacy position them as potentially viable alternatives for aiding diagnostic processes in the future.

## 1. Introduction

The diagnosis of autism represents one of the most pressing bottlenecks in pediatric healthcare. Current clinical assessments rely on extensive behavioral evaluations through standardized instruments such as the Autism Diagnostic Observation Schedule (ADOS) [1,2] and the Autism Diagnostic Interview-Revised (ADI-R) [3], which assess between approximately 20 and 100 behaviors and require several hours of specialized clinical expertise. This resource-intensive process has created an untenable situation: while autism prevalence has risen to affect 1 in 31 children in the United States [4], the average age of diagnosis remains around 5 years [5], extending to 8 years or later in underserved populations [6,7]. These diagnostic delays deny children access to early interventions during the narrow developmental window when behavioral therapies yield the maximum benefit [8,9].

Recent advances in machine learning have demonstrated that behavioral features extracted from brief home videos by humans can lead to diagnostic accuracies exceeding 90% when processed through optimized classifiers [10]. Building on foundational work that focused on clinically accurate but sparse models of 5–10 predictive features [11–14], researchers have successfully validated video-based screening approaches that could theoretically democratize access to diagnostic services and eliminate the need for lengthy evaluations using the current resource-intensive in-clinic approaches [15,16]. These advances led to Washington et al.’s finding that a subset of high-performing non-expert crowdworkers, identified through a stringent crowd filtration process, could reliably extract clinically relevant features from naturalistic videos, achieving 92–98% accuracy when their annotations were fed through validated classifiers [17].

However, the promise of crowdsourced behavioral assessment has been constrained due to operational realities. Washington et al.’s methodology, while a punctuated leap forward for child healthcare, still suffers from scalability issues: from an initial pool of 1107 potential workers on Amazon Mechanical Turk who were evaluated, only 102 (9.2%) passed the rigorous quality control measures necessary to become reliable video annotators [17,18]. This extensive filtering process, requiring multiple training rounds and continuous quality monitoring, presents barriers to large-scale deployment. Moreover, privacy concerns may necessitate video modifications such as face obfuscation and audio pitch shifting for some families who may be hesitant to share raw videos, which can decrease accuracy to as low as 78% [17]. These limitations have the potential to be addressed through fully automated digital diagnostics, which have remained a challenge for the field for many years [19,20].

To address these limitations, there have been several recent efforts to create automated computer vision classifiers for autism [21–33]. While various deep learning approaches have attempted fully automated classification [24–26,31], they consistently achieve lower accuracy (76–85%) than human-in-the-loop methods and additionally, they cannot explain their predictions at the feature level. This interpretability gap makes them unsuitable for clinical deployment, for which understanding the basis of diagnostic decisions is paramount. We seek to understand whether the emergence of multimodal large language models (LLMs) can support automated classification that reaches the performance level of human-in-the-loop approaches. Unlike human-in-the-loop approaches that require extensive human labor by definition, modern multimodal LLMs can directly process and analyze complex behavioral information from video content without human intermediaries [34,35]. These models have the potential to leverage their broad pre-training on diverse visual and linguistic data to understand nuanced social behaviors, eliminating both the bottleneck of human recruitment and the privacy concerns inherent to the human viewing of patient health information (PHI).

The application of LLMs to behavioral assessment represents part of a broader paradigm shift in psychological and mental health diagnostics, with recent work demonstrating that these models can move beyond traditional rating scales to leverage natural language as a richer assessment modality [36]. Studies evaluating LLMs on mental health diagnostic tasks have shown promising results across depression, anxiety, and other conditions, suggesting feasibility for structured diagnostic evaluation in neurodevelopmental disorders [37]. While proposals for responsible development emphasize the need for the careful validation and consideration of potential biases before clinical deployment [38], the emerging capabilities of LLMs to understand complex behavioral patterns from multimodal data offer exciting opportunities for scalable diagnostic tools.

Recent applications of LLMs to autism-related tasks have shown promise, with models demonstrating the ability to match clinical expertise in diagnostic reasoning [39,40], identifying autism-relevant language patterns [41], and leveraging audio-visual information for behavior recognition [27].

In this work, we present the first systematic evaluation of multimodal LLMs as potential replacements for human crowdworkers in autism detection and monitoring. We evaluate seven LLM variants from Google’s Gemini model family—spanning three generations from Gemini 1.5 to 2.5 series—on their ability to extract behavioral features from the same 50 YouTube videos previously annotated by crowdworkers and clinical experts. Each model is asked to respond to a set of behavioral feature questions used by validated machine learning classifiers [10], which have demonstrated high accuracy in prior studies [10,17]. This direct comparison on identical tasks enables us to assess whether general-purpose LLMs can match or exceed the performance of carefully selected and trained human annotators.

Our investigation addresses questions about the viability and mechanisms of LLM-based behavioral assessment. First, we examine whether multimodal LLMs can achieve diagnostic accuracy comparable to human experts, tracking performance evolution across model generations to understand the trajectory of improvement. Second, we investigate how LLMs differ from humans in their assessment strategies—analyzing inter-rater reliability patterns, feature prioritization, and the behavioral cues to which they attend. Third, we identify the factors that determine when LLMs succeed or fail, examining how video characteristics, particularly the presence of autism diagnosis, influence model–human agreement. Finally, through systematic ablation studies, we dissect the contribution of different model components—including structured reasoning, multimodal processing, prompt engineering, and contextual information—to understand the technical requirements for reliable behavioral assessment.

## 2. Related Work

### 2.1. Feature Reduction in Autism Machine Learning Models

The foundation for video-based autism screening emerged from efforts to reduce the complexity of traditional diagnostic instruments to arrive at a quantitative, objective, and scalable diagnostic approach. Wall et al. [11,12] demonstrated through ML analysis of records from use of ADOS and ADI-R that models using 7–8 behavioral features could achieve high accuracy in independent validation datasets, opening up promise for more streamlined approaches. Subsequent refinements produced the LR5 and LR10 classifiers, which use 5 and 10 features to predict autism [13,14]. Such minimally viable feature sets have shown predictive accuracy including with complex cases [42].

### 2.2. Crowdsourced Behavioral Annotation

Building on these reduced feature sets, researchers validated that non-expert crowdworkers could reliably extract diagnostic signals from naturalistic videos. Tariq et al. [10] achieved 88.9% accuracy using approximately 2 min home videos rated by humans in time-frames roughly equivalent to the video lengths, with independent validation maintaining 80% accuracy—demonstrating that brief observations on easy-to-access video data in natural environments could capture clinically meaningful behaviors. Abbas et al. [15] extended this approach by combining video with parent inputs, showing superior performance to traditional screening tools like M-CHAT. Myers et al. [43] provided further validation by demonstrating strong agreement between crowdsourced and expert assessments on toddler videos. Parallel work explored multimodal approaches, with Zhu et al. [32] showing that response-to-name paradigms could effectively identify autism markers in early screening contexts.

However, operational realities limit the scalability of crowdsourced screening. For example, Washington et al.’s comprehensive studies [17–19] identified a bottleneck: only 9.2% of potential workers (102 of 1107) passed the rigorous quality control measures necessary for reliable annotation. Even after extensive training, maintaining annotation quality required continuous monitoring and calibration. Privacy concerns compounded these challenges. For example, face obfuscation and audio pitch shifting, while necessary for protecting participant identity, degraded accuracy from 92% to 78% with combined protections [17]. Researchers explored alternatives like feature replacement methods [44] and structured observation tools [45] to mitigate these limitations, but the tension between scalability, privacy, and accuracy persists. The per-video annotation costs and infrastructure requirements could represent barriers for human-in-the-loop AI approaches to achieve population-scale deployment despite their technical success [20,46]. Now with the advent of LLMs, we have the opportunity to explore ways to balance scaling limits of human-in-the-loop AI approaches with AI solutions.

### 2.3. Evolution of Large Language Models for Behavioral Assessment

The emergence of multimodal foundation models represents a paradigm shift in automated behavioral analysis. Early vision-language models like CLIP [47] and Flamingo [34] demonstrated capabilities in understanding visual content through natural language, though they lacked the specialized reasoning required for clinical assessment. The introduction of models like GPT-4 [48] and particularly the Gemini family [35] marked a breakthrough, combining video understanding with reasoning capabilities that potentially enable better clinical understanding. The Gemini 2.5 series’ incorporation of explicit “thinking” modes [49] enables structured reasoning about complex behavioral patterns—a capability especially relevant for nuanced diagnostic tasks. Recent evidence confirms these capabilities, with Nelson et al. [50] demonstrating that GPT-4o and Gemini 2.0 achieved near-human performance in facial emotion recognition without task-specific training.

Recent applications to autism-specific tasks have also begun to yield solid results. Stanley et al. [39] demonstrated that LLMs can deconstruct the clinical intuition behind autism diagnosis, matching expert reasoning patterns. Jiang et al. [40] showed LLMs functioning as diagnostic copilots in real clinical scenarios, while language-focused approaches successfully identified autism-associated communication patterns [41,51]. Multimodal systems have pushed boundaries further, with audio-visual behavior recognition systems [27] and social reciprocity assessment frameworks [26,52] demonstrating that LLMs can integrate complex behavioral cues across modalities. The broader success of LLMs in healthcare, e.g., from surpassing human experts in predicting neuroscience results [53] to matching physician performance in clinical tasks [54–56], suggests their potential for transforming diagnostic workflows. Medical-specific adaptations like Med-Gemini [57–59] further demonstrate the feasibility of specialized clinical applications.

While numerous fully automated approaches have been developed for autism detection from remote video and audio data, including multimodal fusion methods [60], audio-based classification, and feature-specific prediction models [21,61,62], these remote assessment methods consistently underperform in comparison to human-in-the-loop approaches when analyzing home videos. Specifically focusing on audiovisual classification from naturalistic home settings, Serna-Aguilera et al. [24] achieved 82% accuracy, Kojovic et al. [25] reported 80.9%, and Liu et al. [26] demonstrated 76% accuracy—all below the 92–98% accuracy achieved by crowdworker approaches on similar home video data [17]. Additionally, these deep learning methods function as black-box classifiers that cannot provide feature-level interpretability or explain which specific behaviors drive their predictions. This limitation prevents meaningful comparison with human annotators at the behavioral feature level, which is essential for understanding diagnostic reasoning and ensuring clinical validity. Our work, therefore, focuses on comparing multimodal LLMs with human-in-the-loop approaches, as both can provide interpretable, feature-level assessments that enable direct analysis of how different evaluators prioritize and interpret behavioral markers.

### 2.4. Addressing a Key Gap: Direct Comparison of LLMs and Human Annotators

To our knowledge, no study has directly compared multimodal LLMs against human crowdworkers on identical behavioral annotation tasks. Existing deep learning approaches for autism detection [24,32,63,64] relied on specialized architectures trained specifically for autism classification, requiring a lot of labeled data and offering limited interpretability. These black-box models could not explain their reasoning or identify which behavioral features drove their predictions. In contrast, general-purpose LLMs offer the potential to perform behavioral assessment using their broad pre-training, without task-specific fine-tuning, while providing interpretable feature-level outputs comparable to human annotations.

This comparison is important for determining whether LLMs can address the limitations that have prevented video-based screening from reaching clinical practice. If general-purpose LLMs can match the 92–98% accuracy achieved by carefully selected crowdworkers [17] while eliminating recruitment bottlenecks, training requirements, privacy concerns, and per-video costs, they have the potential to help enable population-scale autism diagnosis. Our work directly addresses this gap by evaluating seven Gemini model variants against both crowdworkers and clinical experts on identical videos, using the same validated classifiers, and analyzing not just accuracy but also agreement patterns, feature prioritization, and the mechanisms underlying their assessments. This comprehensive comparison reveals both the promise and limitations of LLM-based behavioral assessment, informing the path toward scalable diagnostic tools.

## 3. Materials and Methods

**Figure 1.**
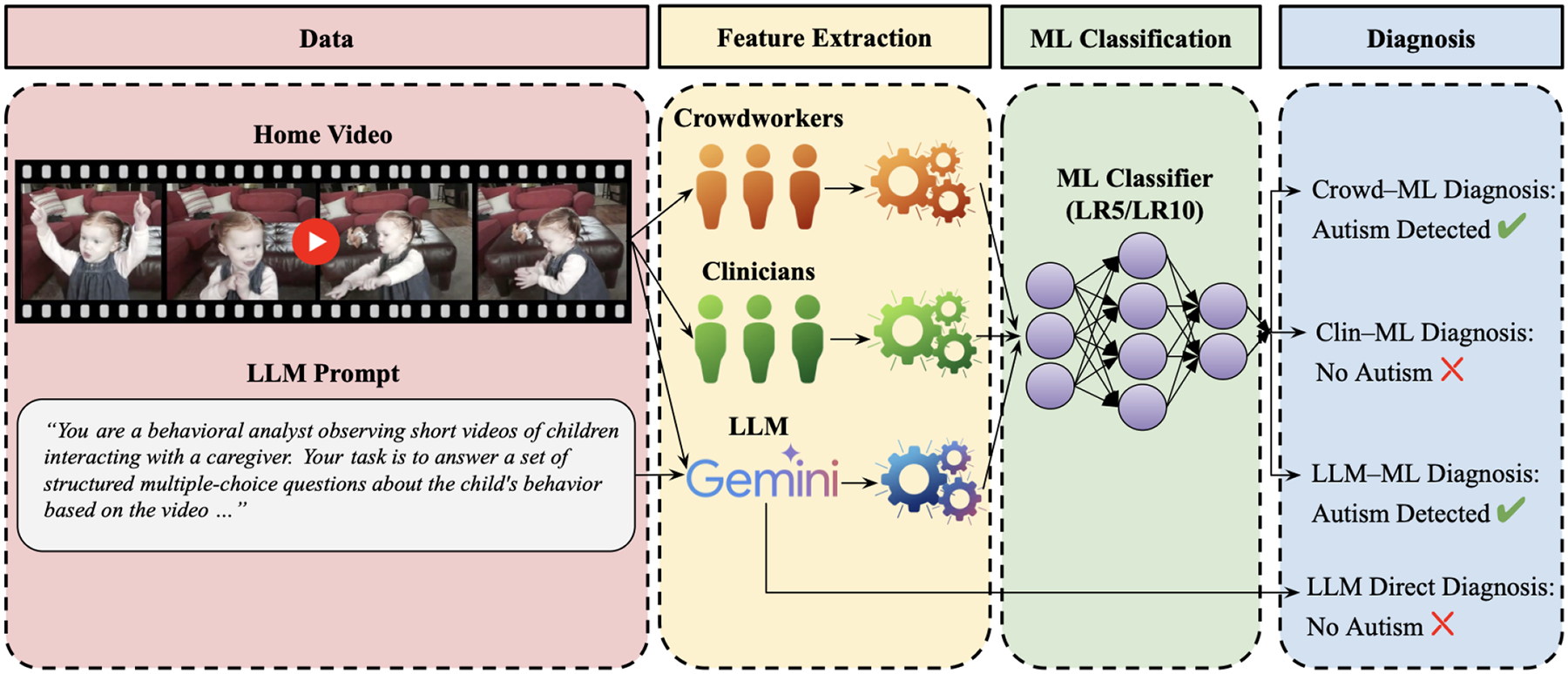
Overview of the experimental pipeline for LLM-based autism detection. The workflow consists of four stages: (1) data collection from home videos with structured prompts, (2) feature extraction through crowdworkers, clinicians, and LLMs to generate behavioral ratings, (3) ML classification using validated LR5/LR10 classifiers, and (4) diagnosis generation through both ML-assisted and direct approaches. The pipeline demonstrates the comparative evaluation framework for assessing LLM performance against human baselines.

Figure 1 illustrates the complete experimental pipeline for evaluating multimodal LLMs against human raters in autism detection, encompassing data collection, feature extraction, machine learning classification, and diagnostic prediction stages.

**Figure 2.**
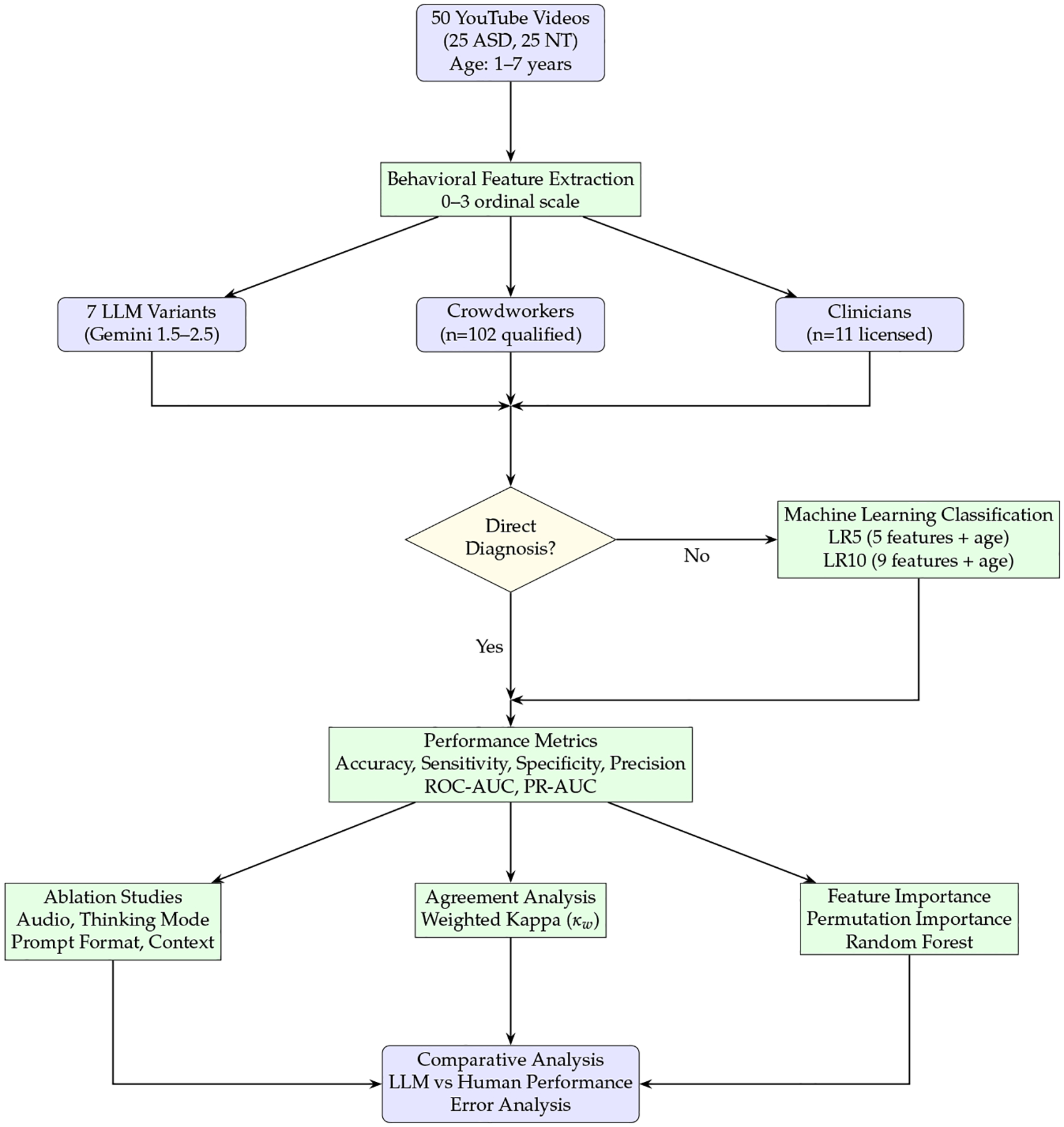
Detailed flowchart of the experimental methodology. The workflow begins with 50 YouTube videos, proceeds through behavioral feature extraction using multiple choice questions, evaluates three groups of raters (LLMs, crowdworkers, and clinicians), and then branches based on direct diagnosis assessment. If direct diagnosis is performed (yes), the workflow bypasses ML classification and proceeds directly to performance evaluation. If not (no), features are processed through machine learning classifiers (LR5/LR10) before performance evaluation. The methodology culminates in a comprehensive analysis including agreement analysis, feature importance analysis, and ablation studies. Purple boxes indicate data/results, green boxes represent processing steps, and the yellow diamond shows the decision point for direct diagnosis assessment.

Figure 2 presents a detailed flowchart of the experimental methodology, illustrating our approach for evaluating multimodal LLMs against human baselines in autism diagnostic assessment. The process begins with data collection from 50 YouTube videos (25 autism, 25 neurotypical), followed by behavioral feature extraction using multiple choice questions on a 0–3 ordinal scale. Three parallel annotation paths are then executed: (1) 7 LLM variants from Gemini 1.5–2.5 series, (2) 102 qualified crowdworkers, and (3) 11 licensed clinicians. The extracted features feed into two assessment approaches: direct diagnosis, for which models provide autism assessments without intermediate classifiers, or machine learning classification using the validated LR5 and LR10 classifiers. Our performance evaluation encompasses multiple metrics (accuracy, sensitivity, specificity, precision, ROC-AUC, PR-AUC), followed by three parallel analysis streams: inter-rater reliability analysis using weighted kappa coefficients, feature importance analysis using permutation importance and random forest methods, and ablation studies examining audio input, thinking mode, prompt format, and behavioral context. Our methodology concludes with a comparative analysis between LLM and human performance, including error analysis and video-level agreement patterns.

### 3.1. Dataset

We utilized the same 50 YouTube videos analyzed by Washington et al. [17], comprising 25 children with parent-reported autism diagnoses and 25 neurotypical controls, balanced by gender (52% male, 48% female). Children ranged from 1 to 7 years (mean: 3.5 years, SD: 1.7 years), representing the typical age range for autism screening. Videos were recorded in naturalistic home settings, averaging 171.3 ± 105.0 s in duration, and captured unstructured play and social interaction scenarios. All videos met three inclusion criteria essential for behavioral assessment: (1) the child’s face and hands were clearly visible, (2) opportunities for social engagement with caregivers or siblings were present, and (3) the child interacted with toys or objects enabling the assessment of play behaviors. This dataset has been extensively validated in prior crowdsourcing studies, with parent-reported diagnoses confirmed through clinical severity ratings [17].

We acknowledge that this curated dataset of YouTube videos may not fully capture the complexities of real-world clinical deployment. The videos were selected for clear visibility of the child’s face and hands, good lighting conditions, and minimal occlusions—conditions that may not always be present in routine clinical or home settings. Real-world applications would need to handle varying video quality, multiple camera angles, partial occlusions, background noise, and cultural differences in behavioral expression and social interaction patterns. However, using this standardized dataset was essential for direct comparison with the established human-in-the-loop baselines from Washington et al. [17], allowing us to isolate the performance differences between LLMs and human annotators under controlled conditions.

### 3.2. Behavioral Feature Extraction

Following Washington et al.’s protocol [17], each video was evaluated using the set of ordinal responses to multiple-choice questions utilized by LR5 and LR10 classifiers. The questions used by each classifier assess three core domains: Language and Communication, Reciprocal Social Interaction, and Stereotyped Behaviors and Restricted Interests. Each question employed a 0–3 ordinal scale, where 0 indicates typical behavior, 1 represents mild differences, 2 signifies moderate differences, and 3 denotes significant differences from typical development. Some behavioral features include numerical suffixes (−1, −2) that distinguish assessments adapted for different developmental levels: −1 for children with emerging language skills and −2 for verbally fluent children. The LLMs received a standardized prompt identifying them as behavioral analysts tasked with scoring observable behaviors in the video (see Appendix A for the complete prompt). Of note, we instructed the models to provide their best assessment even when specific behaviors were not clearly exhibited.

The feature extraction stage transforms raw video observations into quantifiable behavioral markers that serve as inputs to downstream classifiers. The selection of these specific features is grounded in prior work on clinical validation, with each feature capturing distinct aspects of the autism phenotype. Language and Communication features (e.g., echolalia, expressive language, speech patterns) leverage the models’ strong linguistic capabilities from pre-training. Reciprocal Social Interaction features (e.g., eye contact, emotion expression, social overtures) require a nuanced interpretation of non-verbal cues and contextual understanding. Stereotyped Behaviors and Restricted Interests present unique challenges, as they often manifest as subtle repetitive patterns that may be difficult to detect in brief video segments. The structured prompt design ensures consistent interpretation across models, while the ordinal scoring system (0–3) provides a granular assessment beyond binary classification. This multi-dimensional feature extraction approach enables the capture of heterogeneous autism presentations while maintaining clinical interpretability—a key advantage over end-to-end deep learning approaches that operate as black boxes.

### 3.3. Machine Learning Classifiers and Direct Diagnosis

We evaluated model responses using the same two logistic regression classifiers validated in Washington et al. [17]. The LR5 classifier utilizes 5 behavioral features and was trained on ouctomes from administration of ADOS Module 2 (for children with phrase speech) from 1319 children with autism and 70 controls without autism. The LR10 classifier employs 9 features plus age and was trained on outcomes from administration of ADOS Module 3 (for verbally fluent children) from 2870 children with autism and 273 controls without autism. These classifiers represent optimally sparse models that maintain >90% accuracy while using minimal feature sets [13,14]. The specific features for LR5 include: age, stereotyped speech, reciprocal social communication, facial expressions, speech patterns, and eye contact. LR10 extends this set with additional social and communication features, including stereotyped interests, social initiations/overtures, aggression, communicative engagement, and quality of social response.

Beyond classifier-mediated assessment, we evaluated a “direct diagnosis” approach through which models were explicitly asked to determine whether the child in the video showed signs consistent with autism. We tested two prompt formats for this diagnostic question: a binary format (autism/neurotypical) and a three-choice format that included an intermediate “some evidence of autism” option (see Appendix A for exact wording). For the three-choice format, both “some evidence” and “strong evidence” responses were coded as positive for autism diagnosis to maintain binary classification compatibility.

This direct approach was tested in two configurations: (1) with context, where the diagnostic question was presented alongside all the behavioral questions, allowing the model to integrate its behavioral assessments into the diagnostic decision, and (2) without context, where only the diagnostic question was presented in isolation. This design enabled us to assess whether LLMs could leverage behavioral feature patterns for diagnostic reasoning versus making assessments based solely on gestalt impressions.

### 3.4. Model Configurations

We evaluated seven variants from Google’s Gemini model family spanning three generations: Gemini 1.5 (Pro and Flash), Gemini 2.0 (Flash and Flash Lite), and Gemini 2.5 (Pro, Flash, and Flash Lite Preview). All models were accessed through Google Cloud’s Vertex AI API. For efficiency, we implemented a batch-processing approach where all the behavioral questions were sent to each model in a single API call per video, minimizing latency and API costs.

For the 2.5 series models featuring “thinking” mode capabilities [49], we enabled this feature using dynamic budget configuration (thinking_budget = −1), allowing models to allocate computational resources adaptively based on question complexity. The thinking configuration included thought summaries (include_thoughts = True) to capture the model’s reasoning process.

Each model configuration was run five times independently to assess response stability. Maximum output tokens were set to 64,000 to accommodate both the thinking process and final responses. All models received identical multimodal inputs (video with audio). Videos were processed sequentially with exponential backoff for rate limiting (2^*n*^ s for attempt *n*).

### 3.5. Performance Assessment and Human Baselines

Model performance was benchmarked against two human baselines. The first consisted of crowdworkers from the work of Washington et al. [17] who achieved 92–98% accuracy, depending on aggregation method (mean, median, or mode across multiple raters). The second baseline was established through recruited clinical evaluators. We recruited 11 licensed clinicians with expertise in developmental pediatrics or autism assessment to evaluate the same 50 videos. The clinician cohort provided 82 total assessments: one clinician assessed all 50 videos, and the remaining 10 clinicians assessed between 1 and 4 videos each (mean: 7.5 videos per clinician). This resulted in 42 videos having single clinician assessments and 8 videos receiving multiple assessments (3–7 clinicians), with an average of 1.6 clinicians per video. To ensure fair comparison with crowdworker baselines and maintain consistency with the methodology in Washington et al. [17], for videos with multiple clinician assessments, we randomly selected three clinicians and computed mean aggregation to derive consensus scores.

Primary performance metrics included classification accuracy, sensitivity (recall), specificity, precision, ROC-AUC, and PR-AUC. For inter-rater reliability analysis, we employed weighted Cohen’s kappa (*κ*_*w*_), which accounts for the degree of disagreement in ordinal ratings—which is particularly important given the 0–3 scale of behavioral features. Weighted kappa values were computed both within models (across 5 runs) to assess consistency, and between models to evaluate agreement across different architectures.

### 3.6. Statistical Analysis

All metrics are reported as mean ± 95% confidence intervals. For AI models, confidence intervals were computed using t-distribution across five independent runs to assess response stability. For human evaluators, confidence intervals were computed using bootstrap resampling with 10,000 iterations across videos to account for the variable number of raters and heterogeneous sampling structure. For feature importance analysis, we employed both permutation importance (model-agnostic) and random forest feature importance (capturing non-linear interactions), ranking features by their impact on classification accuracy. Video-level agreement was analyzed using Pearson correlation to identify factors influencing model-human consensus.

Performance trajectories across model generations were analyzed using least squares regression to project improvement trends. For ablation studies, we evaluated the contribution of individual components by comparing model performance with and without each feature. Effects are reported as percentage-point differences in accuracy between configurations. Specifically, we examined the following: (1) thinking mode (enabled vs. disabled for 2.5 series models), (2) multimodal input (video + audio vs. video-only), (3) context inclusion (behavioral questions provided with vs. without diagnostic query), and (4) prompt format (binary vs. three-choice response options). These comparisons enabled us to quantify the relative importance of each technical component for reliable behavioral assessment.

During the preparation of this manuscript, the authors used Claude Opus 4.1 for the purposes of drafting and editing text, refining paper structure, formatting results presentations, and improving manuscript clarity and coherence. Additionally, the authors used Gemini 2.5 Flash Image (Nano Banana) to generate some of the icons used in Figure 1 The authors have reviewed and edited the output and take full responsibility for the content of this publication.

## 4. Results

### 4.1. Overall Performance Benchmarking

**Figure 3.**
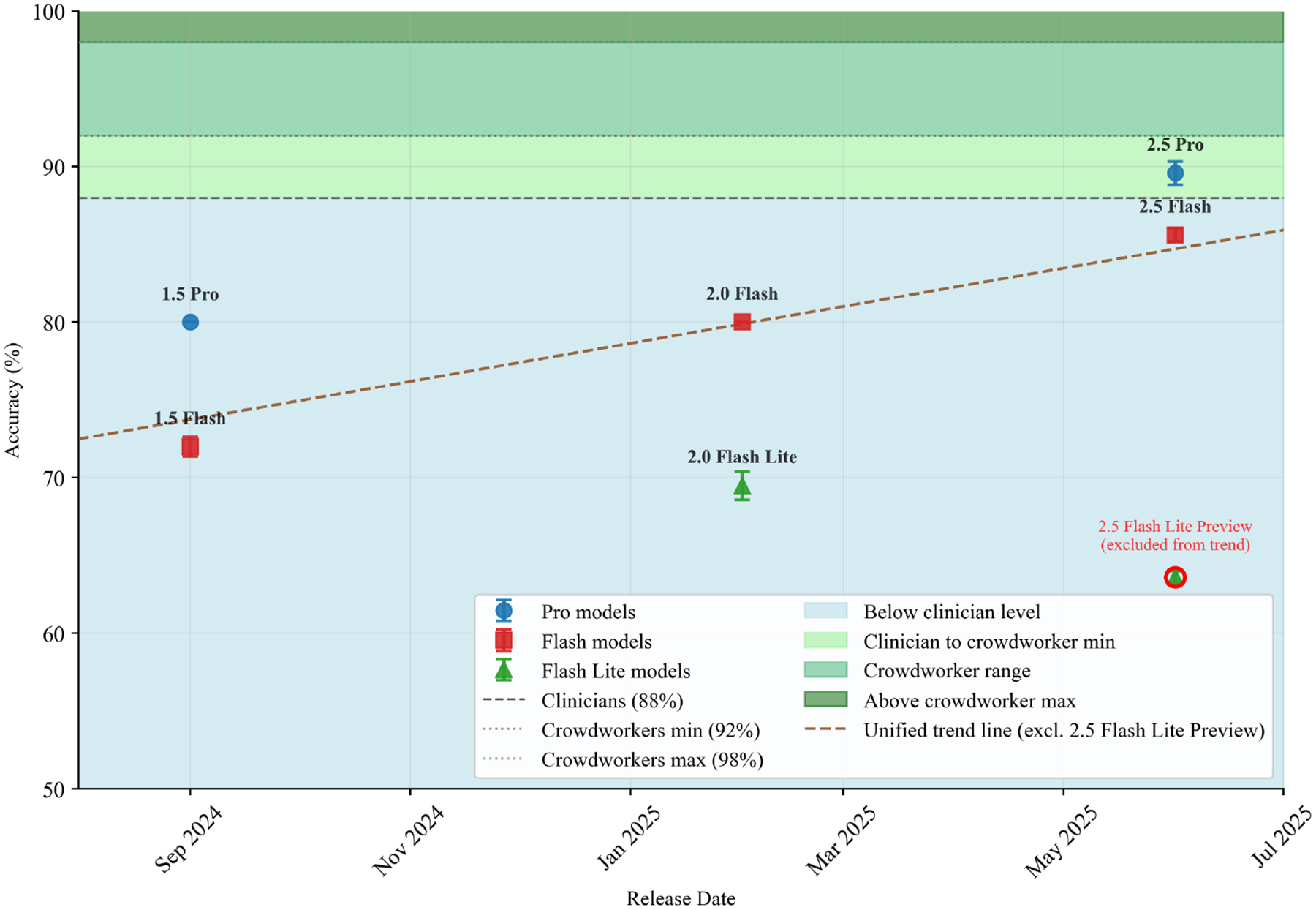
Temporal evolution of Gemini model performance on autism behavioral assessment tasks. The plot shows accuracy progression from September 2024 to June 2025, with a fitted trend line (excluding 2.5 Flash Lite Preview outlier). Human performance benchmarks are indicated by horizontal bands: clinician range (88% baseline) and crowdworker performance (92–98% range). The 2.5 Flash Lite Preview is excluded from the trend analysis due to its inability to process audio, resulting in significantly degraded performance.

We evaluated seven Gemini model variants across three generations on autism behavioral assessment tasks. Table 1 presents performance metrics across all models and human evaluators, while Figure 3 illustrates the temporal evolution of model capabilities. For human benchmarks, we collected clinician evaluations from 11 licensed professionals. Crowdworker performance benchmarks were established from the work of Washington et al. [17], where the reported range (92–98%) reflects different aggregation methods (mean, median, and mode) across multiple raters per video.

Our results demonstrate performance gains across model generations, with accuracy improving from 72.0% (±1.8) in Gemini 1.5 Flash to 89.6% (±2.1) in Gemini 2.5 Pro using the LR5 classifier—representing a 24.4% relative improvement over 15 months of model development. The progression follows a consistent upward trajectory: the 1.5 series achieved 72–80% accuracy, the 2.0 series reached 80%, and the 2.5 series attained 85–90% accuracy. This advancement, visualized by the trend line in Figure 3 (excluding the 2.5 Flash Lite Preview outlier, explained later in this section), shows a least squares regression with a slope of 0.0402 accuracy percentage points per day (*R*^2^ = 0.409, *p* = 0.172). However, this linear projection should be interpreted with caution. The observed trajectory may represent the early phase of a logistic growth curve that will eventually plateau as models approach theoretical performance limits. The temporal progression could follow a logarithmic, rather than linear, scale—a possibility we cannot definitively assess with seven data points spanning 15 months. Therefore, the fitted trend line should be interpreted as characterizing recent historical progress, rather than as a predictive model, acknowledging that the true functional form of advancement may differ from the linear approximation shown.

The performance gap between LLMs and human evaluators has narrowed considerably for the most modern model releases. Gemini 2.5 Pro’s 89.6% accuracy with LR5 falls within the confidence intervals of clinical expert performance (88.0 ± 9.00%)and approaches the lower bound of crowdworker performance (92–98%). The LR10 classifier yields slightly lower performance for LLMs and crowdworkers while demonstrating superior performance for clinicians (clinicians: 98.0 ± 3.00%; crowdworkers: 90–96%; Gemini 2.5 Pro: 87.2 ± 1.10%), indicating that different feature sets may capture complementary aspects of the diagnostic signal. For context, previous fully automated deep learning approaches on different datasets have achieved accuracies ranging from 76–85%. Our best-performing model, Gemini 2.5 Pro at 89.6%, exceeds this range while using general-purpose models without autism-specific training, suggesting that multimodal LLMs may offer advantages over task-specific architectures in both performance and interpretability.

When examining direct diagnosis capabilities—where models provide autism assessments without intermediate classifiers—we observe varying results across the model family. Gemini 2.5 Pro achieves 90.0% (±2.5) accuracy in direct diagnosis, slightly exceeding its classifier-mediated performance. Similarly, 2.5 Flash reaches 85.6% (±1.14) and 2.0 Flash attains 78.0% (±0.00) in direct assessment. Interestingly, some earlier models show higher direct diagnosis accuracy than their classifier-based approaches (1.5 Flash: 82.4% direct vs. 72.0% LR5; 2.0 Flash Lite: 82.7% direct vs. 69.5% LR5).

Gemini 2.5 Flash Lite Preview achieved only 63.6 ± 0.89% accuracy. Despite receiving identical videos with full audio tracks as other models, this model consistently failed to process the audio component. The model repeatedly acknowledged this limitation in its reasoning outputs, stating, “The absence of audio in the clips is a significant limitation, and I’ll have to base my assessments primarily on visual cues, noting when the lack of sound prevents a definitive evaluation,” and “No Audio: This is the most significant hurdle. It completely prevents me from assessing any aspect related to speech, language, vocalizations, echolalia, speech patterns, understanding spoken language.” These self-aware acknowledgments suggest that the model, likely due to its experimental preview stage, could not access or process the audio stream despite its presence in the input. This 22-percentage-point drop from the standard 2.5 Flash model (85.6% with thinking mode) resulted in near-random performance on specificity (47.2 ± 1.79%). Due to this limitation in capturing all necessary behavioral cues, we excluded the 2.5 Flash Lite Preview from our trend line analysis, as it represents a qualitatively different assessment capability, rather than a point on the performance evolution trajectory.

All 2.5 series models were evaluated with thinking mode enabled by default (except 2.5 Flash Lite Preview, which defaults to non-thinking mode and was manually set to thinking mode), allowing them to engage in structured reasoning before generating responses. This capability particularly benefits complex behavioral interpretation, as evidenced by Gemini 2.5 Pro and 2.5 Flash achieving 89.6% and 85.6% accuracy.

The 2.5 Pro model, Google’s state-of-the-art thinking model, maintains high performance across all three evaluation approaches (LR5: 89.6%; LR10: 87.2%; Direct: 90.0%), suggesting robust feature extraction capabilities that generalize across different diagnostic frameworks. Furthermore, the high ROC-AUC scores (Gemini 2.5 Pro: 95.6 ± 0.50% for LR5) indicate excellent discrimination capability, while balanced sensitivity (90.4 ± 2.19%) and specificity (88.8 ± 2.00%) suggest that the model avoids systematic bias toward either over- or under-diagnosis.

Computational requirements reveal trade-offs between performance and efficiency. Processing times range from 14.85 s per video (Gemini 2.0 Flash) to 60.92 s (Gemini 2.5 Pro with thinking), with thinking mode introducing a 3–4× latency increase. The 2.5 Pro model, while achieving the highest accuracy (89.6%), costs $0.113 per video and requires approximately one minute of processing time. In contrast, 2.5 Flash with thinking mode processes videos in 56.29 s at $0.032 per video while maintaining 85.6% accuracy. Thinking mode generates high computational overhead (205,111–308,020 thinking tokens versus 24,000 output tokens), with 2.5 Flash generating the most thinking tokens (308,020) despite a lower cost than Pro. The 2.0 Flash model offers efficiency at 14.85 s and $0.008 per video with 80% accuracy, though without interpretable reasoning. For comparison, human annotation requires 5–10 min at $5–10 per video, making even the slowest LLM configuration 5–10× faster and 44–88× more cost-effective.

Based on these performance evaluations, we selected the three best-performing models—Gemini 2.5 Pro, 2.5 Flash, and 2.0 Flash—to represent the LLM group in subsequent analyses throughout this paper. These models demonstrate the requisite multimodal processing capabilities and achieve performance levels that warrant detailed comparison with human evaluators.

### 4.2. Reliability and Agreement Analysis

We examined inter-rater reliability (IRR) across LLM models and human evaluators to understand consistency patterns in behavioral assessment.

#### 4.2.1. Within-Group vs. Between-Group Agreement

**Figure 4.**
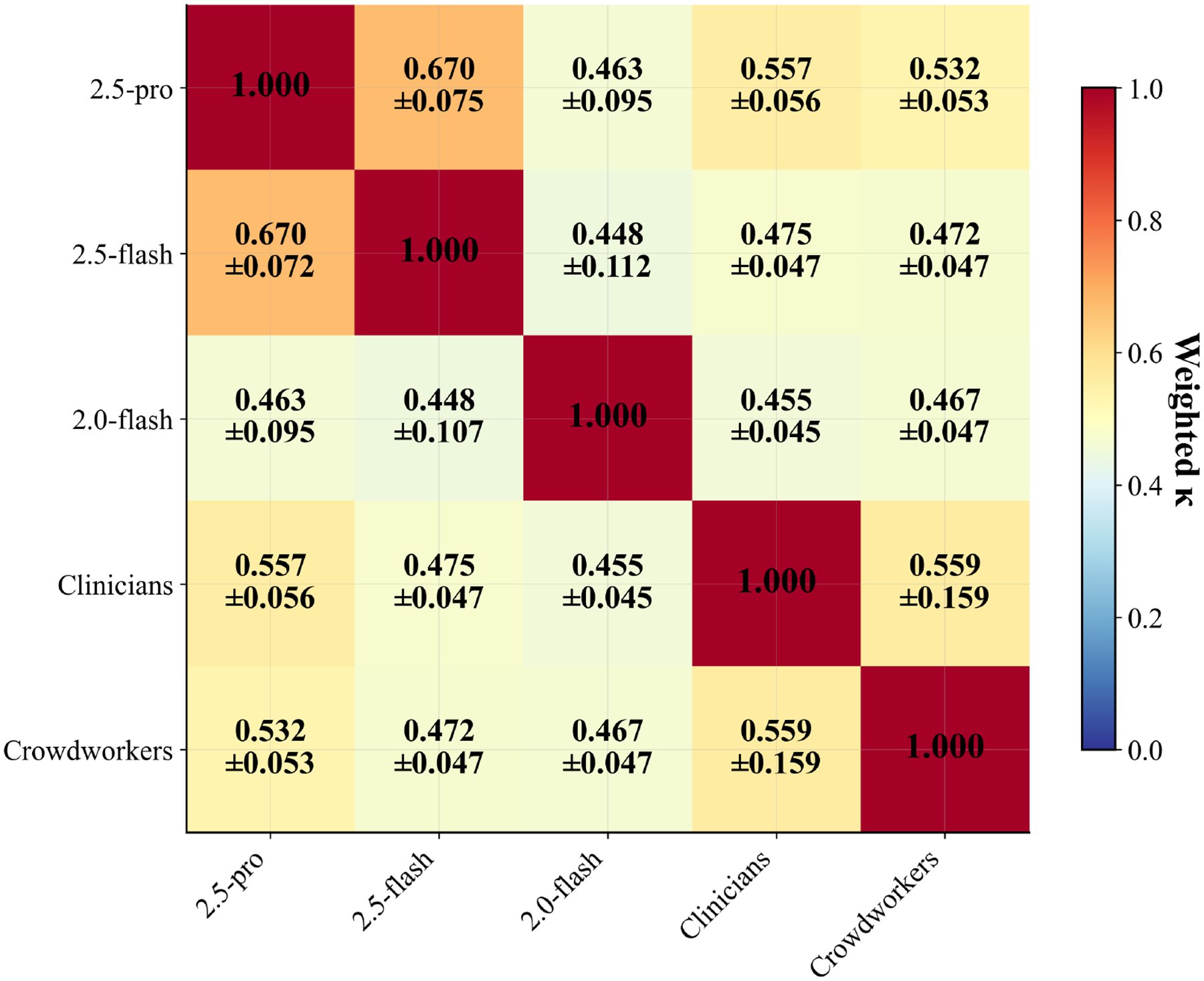
Pairwise inter-rater reliability matrix showing weighted kappa coefficients between all rater groups. Individual LLM models (2.5 Pro, 2.5 Flash, 2.0 Flash) demonstrate near-perfect within-model consistency (diagonal values *κ*_*w*_ > 0.91) but moderate between-model agreement (*κ*_*w*_ = 0.45–0.67).

Figure 4 presents pairwise weighted kappa coefficients across all rater groups. Individual LLM models achieved exceptional within-model consistency when evaluated across multiple runs: Gemini 2.5 Flash demonstrated near-perfect agreement (*κ*_*w*_ = 0.996 ± 0.004, 95% CI: [0.992, 1.000]), followed by Gemini 2.0 Flash (*κ*_*w*_ = 0.989 ± 0.007, 95% CI: [0.982, 0.995]) and Gemini 2.5 Pro (*κ*_*w*_ = 0.917 ± 0.026, 95% CI: [0.889, 0.941]). This consistency exceeds human rater agreement, where crowdworkers achieved *κ*_*w*_ = 0.601 ± 0.163 (95% CI: [0.413, 0.740]) and clinicians showed *κ*_*w*_ = 0.582 ± 0.349 (95% CI: [0.165, 0.862]) using balanced 3-rater sampling per video.

The between-model LLM agreement tells a different story. When comparing responses across the three LLM variants, agreement dropped to *κ*_*w*_ = 0.636 ± 0.050 (95% CI: [0.585, 0.685])—comparable to human inter-rater reliability levels. This pattern is evident in the heatmap (Figure 4), where the off-diagonal LLM-to-LLM cells show moderate agreement (*κ*_*w*_ = 0.448–0.670) despite each model’s internal consistency. This shows that different models have developed distinct assessment strategies in how they approach behavioral assessment and interpret autism markers.

To understand these divergent strategies, we analyzed reasoning outputs from the thinking mode across videos where models both agreed and disagreed on diagnoses. Table 2 presents eight exemplar videos demonstrating that models employ different assessment strategies regardless of their final conclusions. In disagreement cases, such as Video V1, Gemini 2.5 Pro’s atypical behavior-focused approach (“Name called, and no response. That’s a red flag”; “The arm flapping is a classic stim”) led to an autism diagnosis, while 2.5 Flash’s strength-based assessment (“Clear positive social interaction—Logan hugs a caregiver”; “joint attention and shared activity”) led to the conclusion of neurotypical development.

Remarkably, even when models reached identical conclusions, their assessment strategies remained distinct. For Video V3 (both concluded neurotypical), 2.5 Pro emphasized social performance (“he is not just singing; he is performing… showing social awareness”), while 2.5 Flash systematically evaluated diagnostic criteria (“run through the typical indicators for ASD”). Similarly, for Video V15 (both concluded autism), 2.5 Pro provided temporal behavioral mapping with timestamps, while 2.5 Flash organized observations by diagnostic categories. These consistent strategic differences across both agreement and disagreement cases demonstrate that models have developed stable, distinct assessment frameworks: 2.5 Pro employs a clinical, pattern-recognition approach focusing on diagnostic markers, while 2.5 Flash uses a developmental, contextualization approach emphasizing overall functioning.

Agreement between LLMs and human raters revealed moderate concordance, with LLM-clinician agreement ranging from *κ*_*w*_ = 0.455 to 0.557 and LLM-crowdworker agreement from *κ*_*w*_ = 0.467 to 0.532. Notably, Gemini 2.5 Pro showed the highest agreement with clinicians (*κ*_*w*_ = 0.557 ± 0.056), suggesting that its reasoning processes may better align with clinical expertise. The clinician–crowdworker agreement (*κ*_*w*_ = 0.559 ± 0.165) established a human baseline that LLMs approach but do not exceed, indicating that model-human disagreement falls within the range of human inter-rater variability.

#### 4.2.2. Feature-Level and Domain-Level Reliability

**Figure 5.**
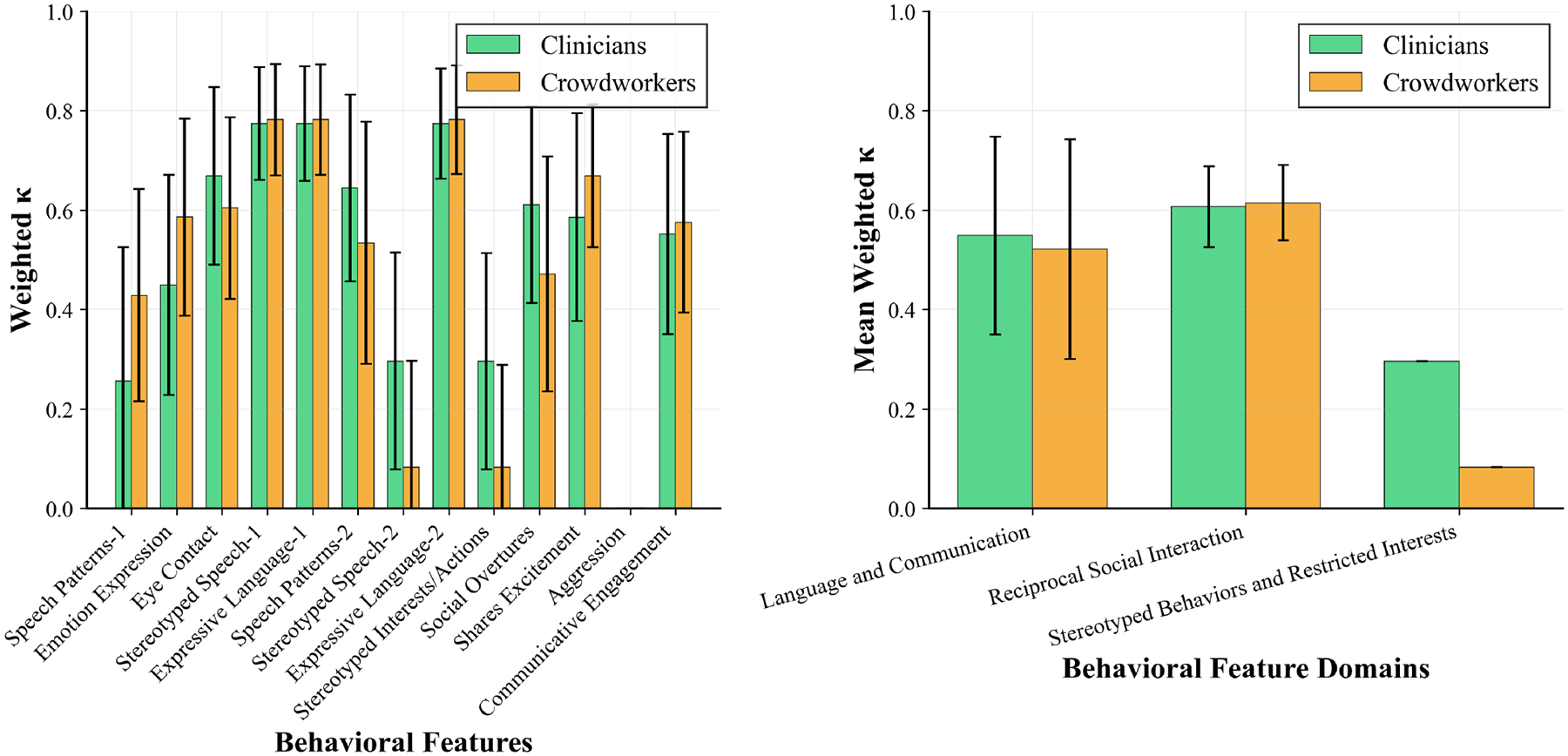
Feature-level and domain-level inter-rater agreement of Gemini 2.5 Pro with clinicians and Gemini 2.5 Pro with crowdworkers. (**Left**) Individual behavioral features show high variability in agreement, with language-related features demonstrating the highest concordance. (**Right**) Domain-level aggregation reveals systematic differences, with Stereotyped Behaviors showing notably lower reliability than the Language and Social domains.

Figure 5 decomposes agreement between our best-performing model (Gemini 2.5 Pro) and human raters at both the feature and domain levels.

Language-related features showed the most consistent high agreement. Both Expressive Language items achieved strong concordance with both groups (*κ*_*w*_ = 0.77–0.78 for both clinicians and crowdworkers). However, Stereotyped Speech features revealed divergence: one item showed excellent agreement (*κ*_*w*_ = 0.774 with clinicians, *κ*_*w*_ = 0.782 with crowdworkers), while the other one demonstrated poor concordance (*κ*_*w*_ = 0.296 with clinicians, *κ*_*w*_ = 0.083 with crowdworkers). Similarly, Speech Patterns features showed disparate reliability: one version achieved moderate agreement (*κ*_*w*_ = 0.664 with clinicians, *κ*_*w*_ = 0.534 with crowdworkers), while another one showed poor concordance (*κ*_*w*_ = 0.256 with clinicians, *κ*_*w*_ = 0.429 with crowdworkers).

Social interaction features revealed complex patterns between rater groups. Eye Contact showed moderate-to-good agreement with both groups (*κ*_*w*_ = 0.669 with clinicians, *κ*_*w*_ = 0.604 with crowdworkers). Shares Excitement demonstrated higher agreement with crowdworkers (*κ*_*w*_ = 0.669) than clinicians (*κ*_*w*_ = 0.586), while Social Overtures showed the opposite pattern (*κ*_*w*_ = 0.611 with clinicians vs. *κ*_*w*_ = 0.472 with crowdworkers). Emotion Expression and Communicative Engagement showed moderate agreement with both groups (*κ*_*w*_ = 0.449–0.586 and *κ*_*w*_ = 0.552–0.576, respectively).

Stereotyped behavior features showed the poorest reliability. Stereotyped Interests/Actions item demonstrated near-random agreement (*κ*_*w*_ = 0.296 with clinicians, *κ*_*w*_ = 0.083 with crowdworkers), while Aggression could not be analyzed as Gemini 2.5 Pro showed zero variation, assigning a score of 0 to all videos, preventing meaningful agreement calculation for this feature.

Domain-level aggregation (Figure 5, right panel) revealed that Gemini 2.5 Pro achieved comparable agreement with both human groups for Language and Communication features (clinicians: *κ*_*w*_ = 0.549, crowdworkers: *κ*_*w*_ = 0.522) and Reciprocal Social Interaction features (clinicians: *κ*_*w*_ = 0.607, crowdworkers: *κ*_*w*_ = 0.615). However, the Stereotyped Behaviors and Restricted Interests domains showed poor agreement (clinicians: *κ*_*w*_ = 0.296, crowdworkers: *κ*_*w*_ = 0.083).

### 4.3. Feature Attribution and Interpretability

To understand the mechanisms underlying model predictions, we analyzed feature importance across LLMs and human raters using both permutation importance and random forest methods.

**Figure 6.**
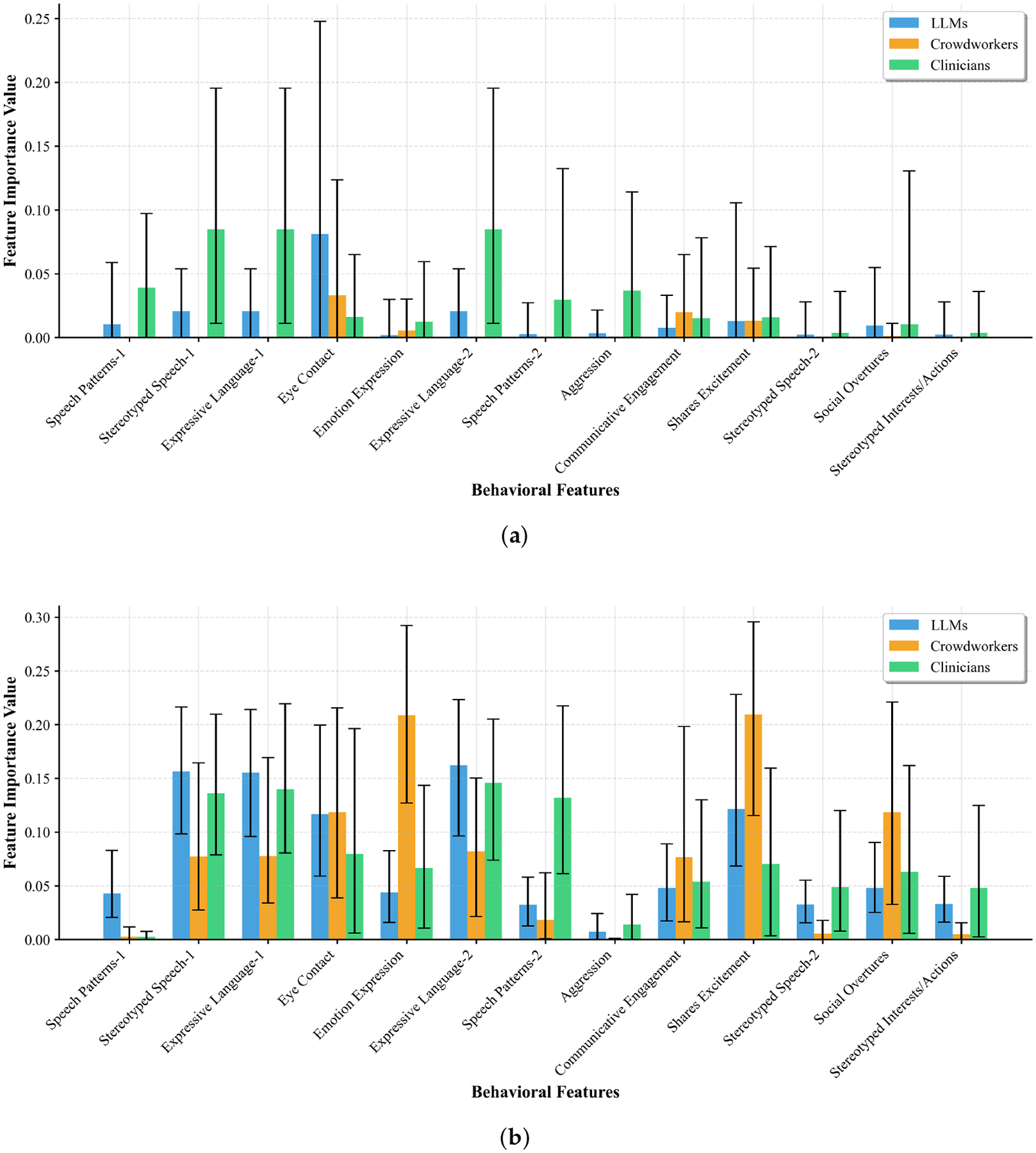
Feature importance analysis across LLMs, crowdworkers, and clinicians using (**a**) permutation importance measuring direct causal impact and (**b**) random forest capturing non-linear relationships. Error bars represent 95% confidence intervals from bootstrap analysis. (**a**) Permutation importance; (**b**) random forest importance.

Figure 6 presents feature importance values computed through permutation importance (Figure 6a) and random forest analysis (Figure 6b), revealing differences in how LLMs and humans prioritize behavioral features. Table 3 synthesizes these findings by ranking the top-5 features for each group using consensus rankings across both methods.

The LLM models demonstrate a clear prioritization of language-related features, with Stereotyped Speech and both Expressive Language items occupying three of their top five positions. This language-centric approach achieves particularly high importance values in the random forest analysis, where Expressive Language emerges as the top feature with importance value of 0.162 ± 0.063. Notably, Eye Contact, while showing the highest permutation importance for LLMs (0.081 ± 0.124), drops to fifth in random forest analysis (0.117 ± 0.070), suggesting that its impact may be more direct, rather than capturing complex interactions.

Crowdworkers exhibit a different assessment strategy, prioritizing social interaction features. Shares Excitement ranks as their most important feature overall, achieving the highest random forest importance (0.210 ± 0.090) among all features across all groups. Emotion Expression shows similarly high importance (0.209 ± 0.083), representing a dramatic 3.7-fold difference compared to LLMs’ valuation of this feature (0.044 ± 0.033 in random forest). This suggests crowdworkers rely heavily on emotional cues that LLMs systematically underweight.

Clinicians present a balanced approach that bridges the LLM and crowdworker strategies. They prioritize language features (Expressive Language: 0.146 ± 0.066 and 0.140 ± 0.069, Stereotyped Speech: 0.136 ± 0.065 in random forest) similar to LLMs, while also incorporating Speech Patterns (0.132 ± 0.078) as their fourth most important feature.

The comparison between permutation and random forest methods reveals both consistencies and divergences in feature attribution. While both methods generally agree on top features within each group, they capture different aspects of feature contribution. Permutation importance, measuring direct causal impact through feature corruption, produces relatively sparse importance distributions with many features showing near-zero values. Random forest importance, capturing non-linear relationships and feature interactions, yields more distributed importance values with clearer differentiation between features.

This comparison is particularly revealing for features like Eye Contact, which shows the highest permutation importance for LLMs (0.081) but moderate random forest importance (0.117), suggesting its contribution is more direct than interactive. Conversely, Communicative Engagement shows minimal permutation importance for crowdworkers (0.020) but high random forest importance, indicating this feature’s value emerges through complex interactions with other behavioral markers.

The differences in importance magnitudes between methods—with random forest values often 5–10 times higher than permutation values—reflect their different measurement approaches. Permutation importance’s lower values suggest that individual feature corruption has a modest impact on model predictions, while random forest’s higher values indicate features contribute more through their participation in decision trees.

### 4.4. Video Characteristics and Error Analysis

To understand the factors influencing model-human agreement and identify potential sources of error, we analyzed the relationship between video-level characteristics and inter-rater agreement across all 50 videos. Specifically, we computed the mean pairwise weighted kappa between each of the three top-performing LLMs (Gemini 2.5 Pro, 2.5 Flash, and 2.0 Flash) and each human group (crowdworkers and clinicians), yielding a composite measure of LLM-human consensus for each video.

#### 4.4.1. Distribution of Agreement and Uncertainty

**Figure 7.**
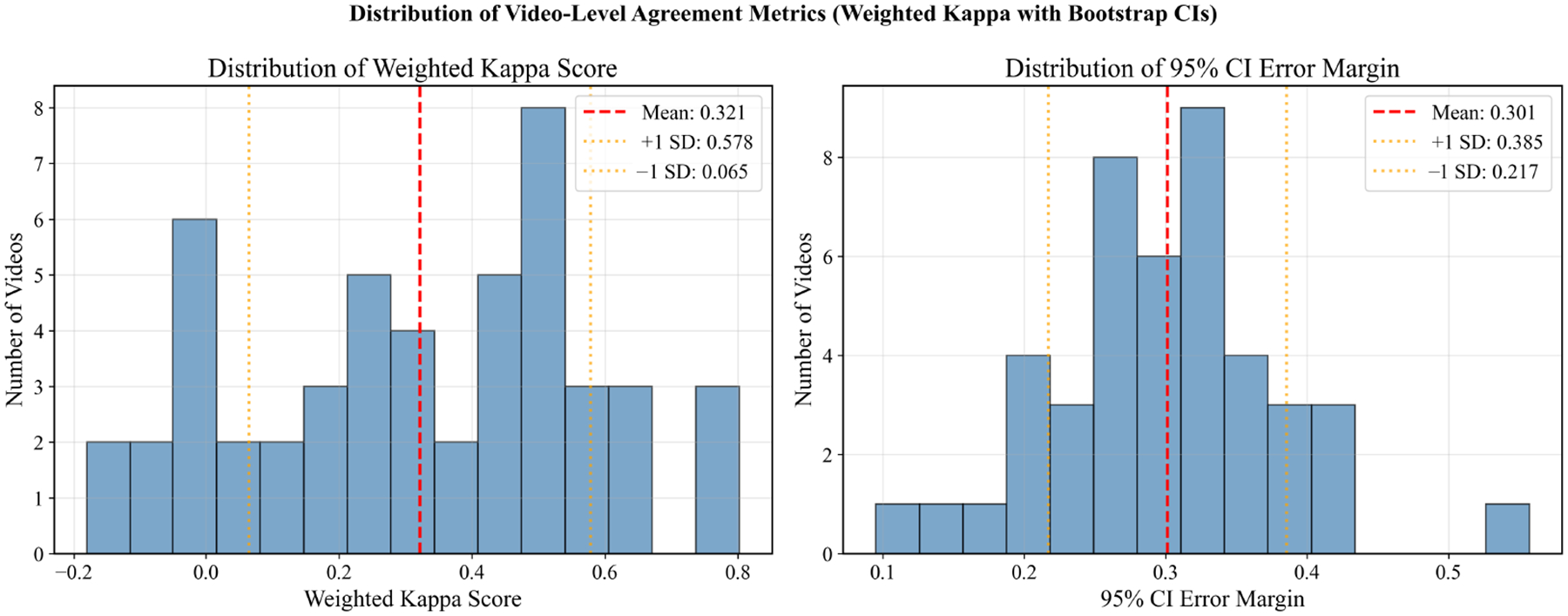
Distribution of video-level LLM-human agreement metrics across 50 videos, computed as the mean pairwise weighted kappa between each LLM and each human group. **Left**: distribution of weighted kappa scores showing high variability (mean *κ*_*w*_ = 0.321 ± 0.257). **Right**: distribution of 95% CI error margins indicating heterogeneous uncertainty levels.

Figure 7 presents the distribution of video-level agreement metrics across our dataset. The weighted kappa scores exhibit high variability (mean *κ*_*w*_ = 0.321 ± 0.257, range: −0.181 to 0.802), indicating that while models achieve consensus on certain videos, they disagree on others. The distribution’s wide spread and inclusion of negative agreement values suggest that model consistency is highly video-dependent, rather than uniformly reliable. The corresponding distribution of 95% confidence interval error margins (mean = 0.300 ± 0.084) further underscores this heterogeneity, with some videos yielding highly uncertain agreement estimates.

#### 4.4.2. Diagnosis as the Primary Driver of Agreement

**Figure 8.**
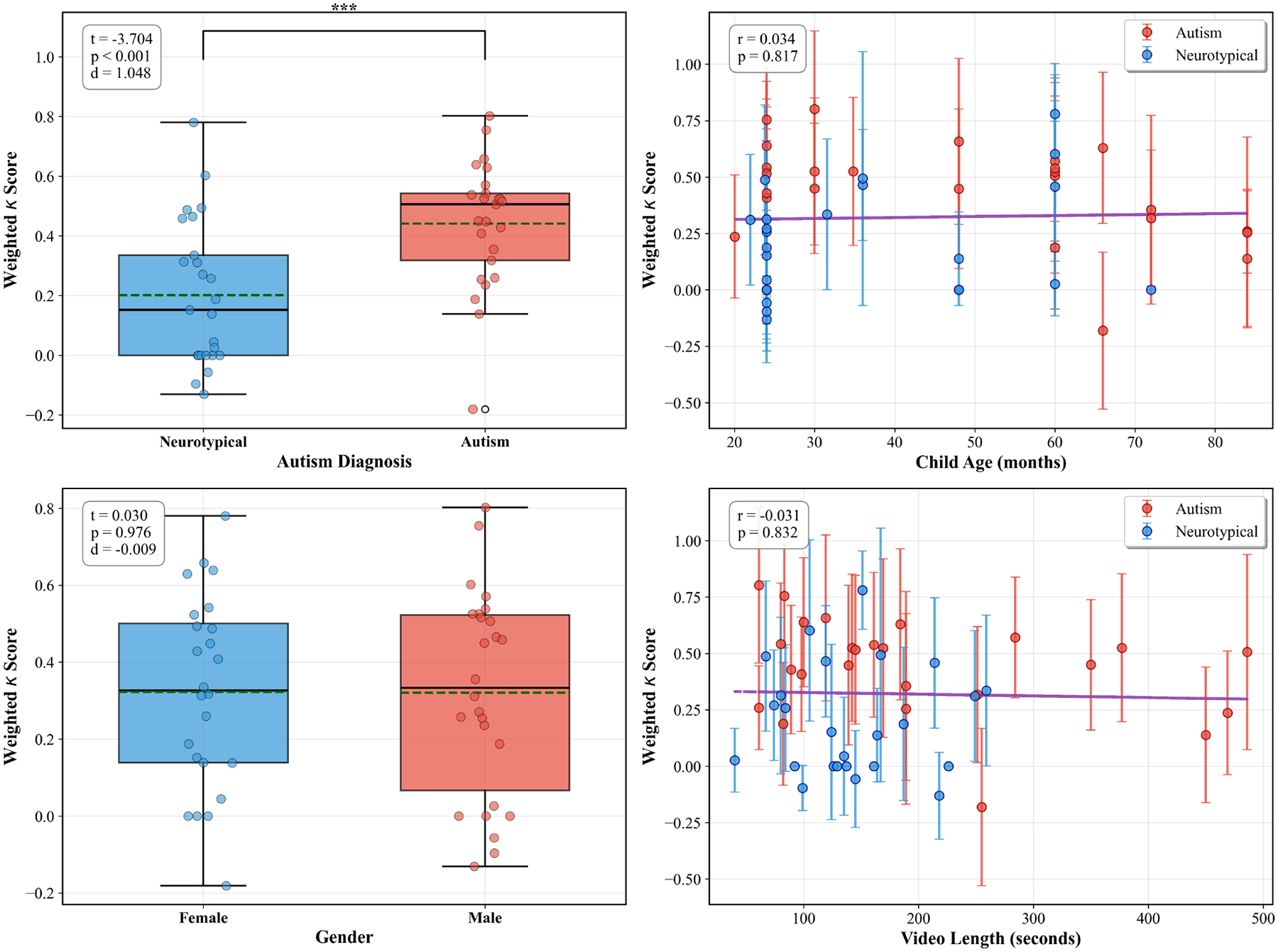
Relationship between video characteristics and model agreement (weighted kappa). Box plots show comparisons for categorical variables (autism diagnosis and gender) with *t*-test statistics, while scatter plots show correlations for continuous variables (age and video length). Error bars represent 95% bootstrap confidence intervals.

Among all video characteristics examined, the presence of an autism diagnosis showed a meaningful effect on model agreement (*t*(48) = −3.704, *p* < 0.001, Cohen’s *d* = 1.048), as illustrated in Figure 8. This large effect size indicates that videos from children with autism (*M* = 0.441, *SD* = 0.213) demonstrated higher model-human agreement than neurotypical videos (*M* = 0.202, *SD* = 0.243). The box plots reveal this separation, with autism cases showing both higher median agreement and less variability.

In contrast, the other characteristics showed no meaningful relationships with agreement levels. Gender had no significant effect (*t*(48) = 0.030, *p* = 0.976, Cohen’s *d* = −0.009), with nearly identical agreement for female (*M* = 0.323, *SD* = 0.252) and male videos (*M* = 0.320, *SD* = 0.266). Similarly, neither child age (*r* = 0.034, *p* = 0.817) nor video length (*r* = −0.031, *p* = 0.832) showed meaningful correlations with agreement levels.

#### 4.4.3. Case Analysis: High vs. Low Agreement Videos

To understand the qualitative factors driving agreement patterns, we examined videos at the extremes of the agreement distribution. The three highest agreement videos (*κ*_*w*_ > 0.755) comprised two autism cases and one neurotypical case, while the lowest agreement videos (*κ*_*w*_ < −0.096) included one autism case and two neurotypical cases.

##### High-Agreement Cases:

**Figure 9.**
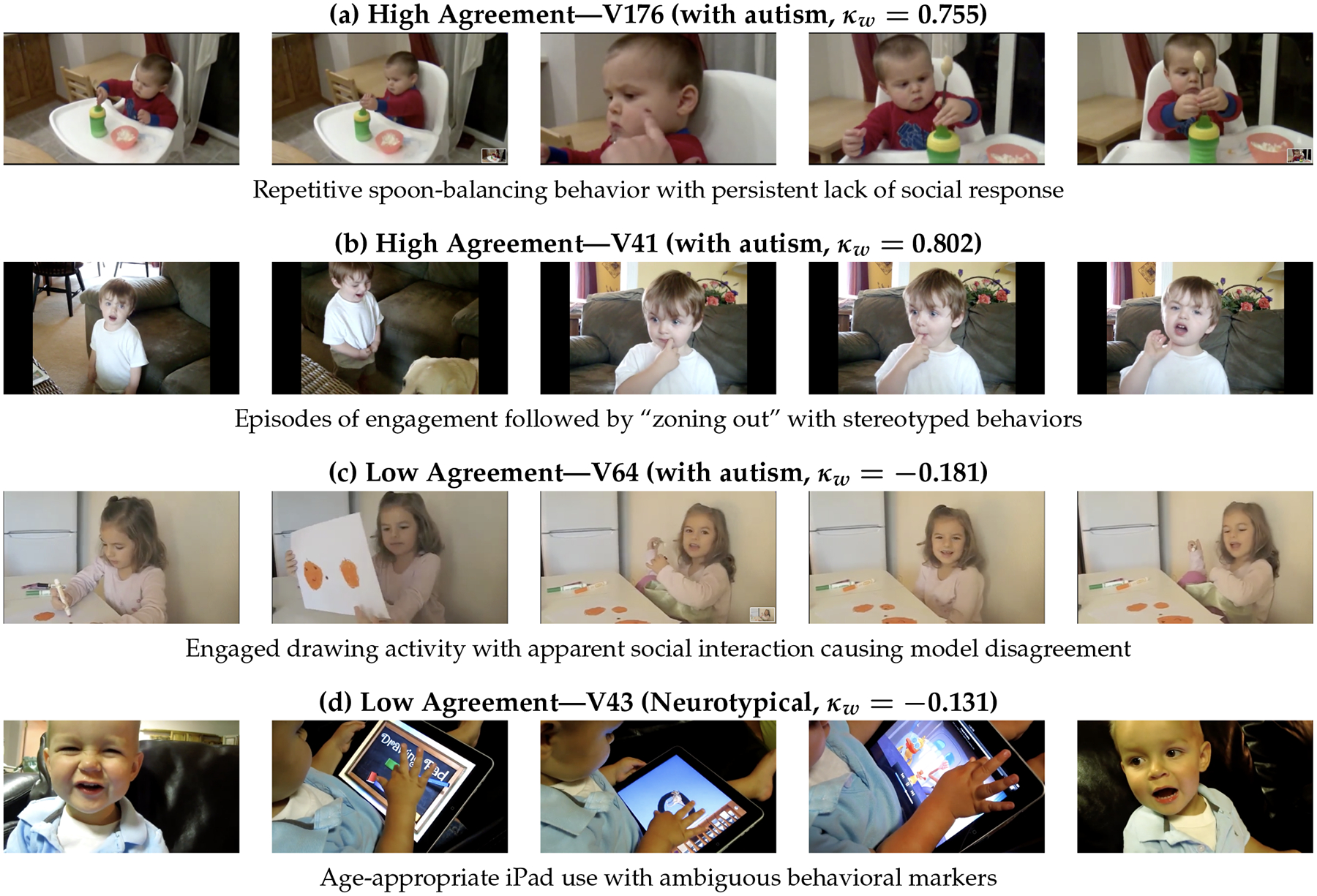
Representative frames from videos with highest and lowest model agreement. High agreement videos (**a**,**b**) show unambiguous behavioral markers that models consistently identify. Low agreement videos (**c**,**d**) present subtle or age-ambiguous behaviors that trigger divergent model interpretations.

An analysis of model reasoning reveals that LLM–human consensus emerges when behavioral markers are unambiguous. In video V176 (*κ*_*w*_ = 0.755, with autism), all models consistently identified the child’s repetitive spoon-balancing behavior, complete absence of verbal communication, and persistent lack of response to name-calling as clear autism indicators (Figure 9a). Similarly, in V39 (*κ*_*w*_ = 0.780, neurotypical), models unanimously recognized age-appropriate conversational skills, sustained eye contact, and reciprocal social engagement. The high agreement on V41 (*κ*_*w*_ = 0.802, with autism) stemmed from the consistent detection of repetitive vocalizations (“uh uh uh”), episodes of “zoning out,” and limited functional communication (Figure 9b).

##### Low-Agreement Cases:

Disagreement primarily arose in videos presenting ambiguous or borderline behaviors. In V64 (*κ*_*w*_ = −0.181, with autism), Gemini 2.5 Pro interpreted the child’s behavior as neurotypical based on apparent conversational ability and social engagement, while 2.5 Flash identified subtle echolalia and atypical language patterns consistent with autism (Figure 9c). This divergence suggests that models may have different sensitivity thresholds for detecting subtle autism markers. The low agreement on V43 (*κ*_*w*_ = −0.131, neurotypical) resulted from differing interpretations of age-appropriate behavior, with models disagreeing on whether certain behaviors represented developmental delays or normal variation for 2-year-olds (Figure 9d).

#### 4.4.4. Systematic Error Pattern Analysis

Analysis of videos with systematic disagreement (negative weighted kappa) revealed failure cases. Models exhibited deterministic behavior—approximately 95% of features showed zero variance across five independent runs. The most severe disagreement (V64, *κ*_*w*_ = −0.181, 66-month-old female with ASD) exposed complete diagnostic failure. Gemini 2.5 Pro assigned all zeros across all five runs with perfect consistency (std = 0.00), stating “Eye contact? Natural and appropriate… I don’t think so [autism]. Score: 0,” entirely missing the autism diagnosis. Conversely, 2.5 Flash assigned identical high severity ratings across all runs (std = 0.00) despite generic thinking outputs (“I’ll watch… I’ll score… Let’s get started”) lacking specific behavioral observations.

Most concerning was the thinking-rating disconnect. In V94 (neurotypical 24-month-old), Flash explicitly concluded “the child shows no signs of autism… Score 0” in its reasoning yet consistently rated social initiation at maximum severity across all runs—a direct contradiction between verbal reasoning and numerical output. V51 revealed diagnostic misinterpretation, with Pro consistently pathologizing advanced knowledge: “The capital quiz is the defining feature… clear pattern emerges,” diagnosing autism (q31 = 1) in all runs. Notably, while Flash showed perfect consistency, Pro demonstrated high variance for some features, suggesting architectural differences in decision stability.

### 4.5. Ablation Studies and Component Analysis

To understand the individual contributions of key experimental factors to model performance, we conducted systematic ablation studies examining four components: audio input processing, thinking mode activation, prompt format design, and behavioral context inclusion. These experiments reveal the essential ingredients for optimal LLM-based behavioral assessment and provide insights into the mechanisms underlying model performance.

#### 4.5.1. Effect of Audio Input

**Figure 10.**
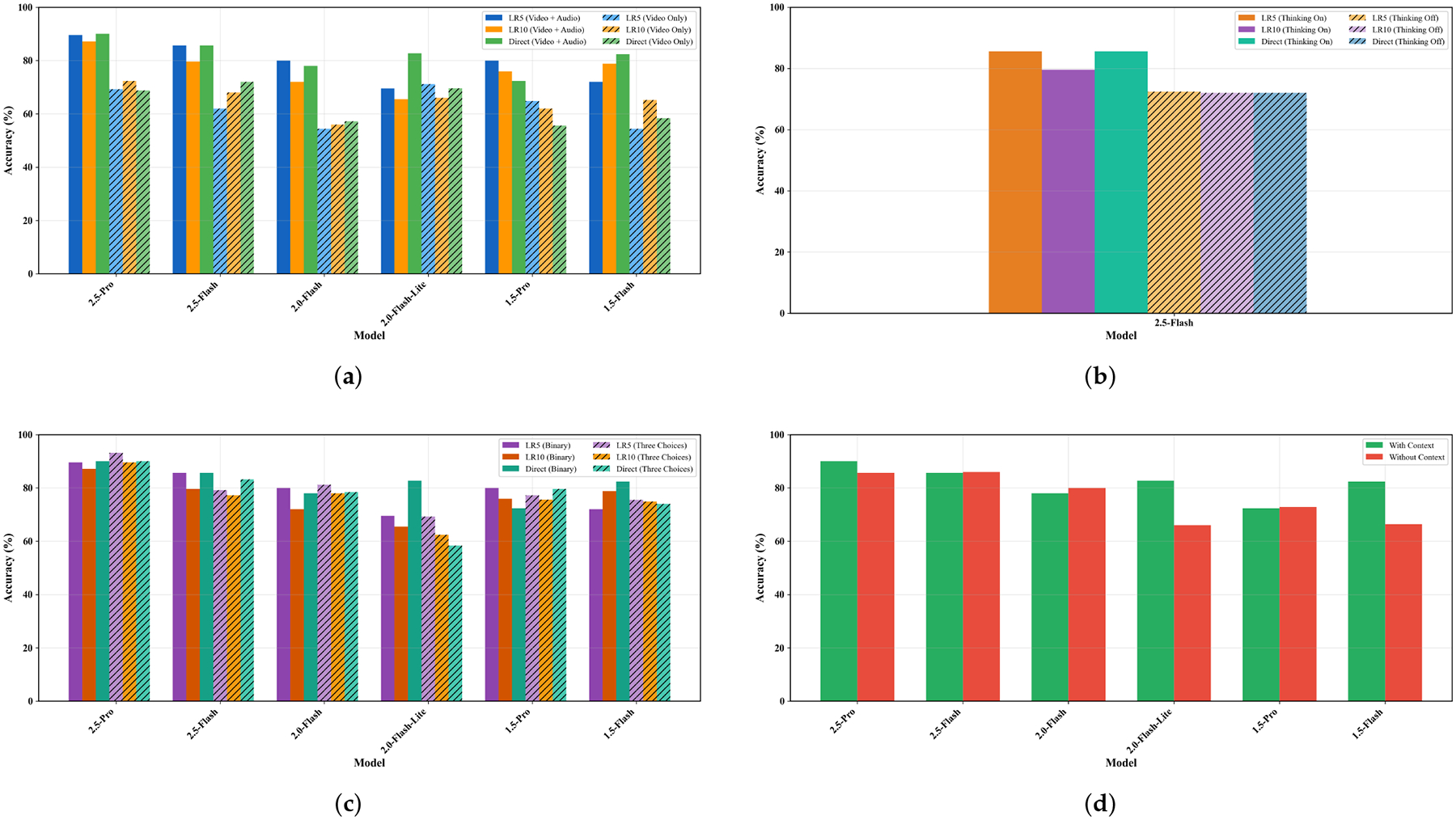
Ablation study results: (**a**) effect of audio input, (**b**) effect of thinking mode, (**c**) effect of prompt format, (**d**) effect of context (direct diagnosis).

Figure 10a demonstrates the profound impact of audio information on diagnostic accuracy, with all models experiencing performance degradation when restricted to visual information alone. The removal of audio channels results in accuracy drops ranging from 11.6% to 25.6% across different model-classifier combinations.

The most dramatic degradation occurs in the 2.0 Flash model, where LR5 accuracy drops from 80.0% to 54.4% (−25.6 percentage points), representing a 32% relative performance loss. Similarly, 2.5 Flash drops from 85.6% to 62.0% (−23.6 percentage points) for LR5, while 2.5 Pro falls from 89.6% to 69.2% (−20.4 percentage points). Even the direct diagnosis approach, which bypasses feature-based classification, shows severe degradation, with 2.5 Pro dropping from 90.0% to 68.8% (−21.2 percentage points).

Interestingly, the 2.0 Flash Lite model presents an anomalous pattern, showing minimal change or even slight improvement without audio (LR5: 69.5% to 71.2%, +1.7 percentage points), suggesting this model may have already adapted to limited audio input. However, its baseline performance of 69.5% aligns closely with the degraded performance of audio-deprived models, validating that ~70% accuracy represents the practical ceiling for video-only assessment. This finding gains additional context when considered alongside the 2.5 Flash Lite Preview’s performance—which consistently reported inability to process audio channels—indicating the convergence of intentionally audio-deprived models with the Flash Lite Preview’s baseline.

#### 4.5.2. Effect of Thinking Mode

The activation of thinking mode in Gemini 2.5 Flash produces performance improvements across all evaluation metrics (Figure 10b). With thinking enabled, the model achieves 85.6% accuracy on LR5 classification, compared to 72.4% without thinking—a 13.2 percentage point improvement representing an 18.2% relative gain.

This enhancement extends consistently across classifiers: LR10 improves from 72.0% to 79.6% (+7.6 points), while direct diagnosis increases from 72.0% to 85.6% (+13.6 points). The magnitude of these improvements effectively bridges the performance gap between model generations—a 2.5 Flash model without thinking performs comparably to 1.5 series models, while the thinking-enabled version approaches the 2.5 Pro performance ceiling.

#### 4.5.3. Effect of Prompt Format

The impact of prompt format shows heterogeneity across models (Figure 10c). For 2.5 Flash, the binary format outperforms the three-choice alternative (LR5: 85.6% vs. 79.2%, −6.4 percentage points). Meanwhile, 2.5 Pro shows the opposite pattern, with the three-choice format yielding marginally better LR5 performance (93.2% vs. 89.6%, +3.6 percentage points, though this doesn’t extend to direct diagnosis (90.0% for both formats).

The most extreme response occurs in 2.0 Flash Lite’s direct diagnosis, where the three-choice format degrades performance from 82.7% to 58.4% (−24.3 percentage points).

#### 4.5.4. Effect of Behavioral Context

The inclusion of behavioral context through the feature-extraction questions reveals a clear generational progression in model autonomy (Figure 10d). Earlier models show strong context dependency: 1.5 Flash improves from 66.4% to 82.4% with context (+16.0 percentage points), while 2.0 Flash Lite gains similarly from 66.0% to 82.7% (+16.7 percentage points).

In contrast, the 2.5 series models demonstrate context independence. 2.5 Flash shows virtually no change (85.6% with context vs. 86.0% without, −0.4 percentage points), while 2.5 Pro shows only modest improvement with context (90.0% vs. 85.6%, +4.4 percentage points). The 2.0 Flash model shows a slight degradation with context (78.0% vs. 80.0%, −2.0 percentage points).

## 5. Discussion

This study presents the first evaluation of multimodal large language models’ ability to provide accurate feature measures useful for predicting autism given short naturalistic videos. Human-in-the-loop AI has shown promise in clinical-grade performance in digital autism diagnostics, with crowdsourcing approaches like those of Washington et al. [17] achieving 92–98% accuracy but requires human resources that could ultimately be difficult to scale. Similarly, recent efforts to automate the process using computer vision and traditional machine learning show promise but still require humans in the loop. The advancements of multimodal LLMs has enabled us to test the hypothesis that LLMs could, given proper engineering and tuning, lead to new advances in automation of diagnostic approaches.

### 5.1. Overall Performance Benchmarking

Our results (Figure 3 and Table 1) demonstrate a clear trajectory: the newer the LLM generation, the better the performance. Accuracy improved from 72.0% in Gemini 1.5 Flash to 89.6% in Gemini 2.5 Pro using the LR5 classifier—representing a 24.4% relative improvement over 15 months of model development. This progression follows a consistent upward trajectory visualized with the trend line in Figure 3, showing a least squares regression with a slope of 0.0402 accuracy percentage points per day. While Gemini 2.5 Pro’s 89.6% accuracy does not quite reach the performance of human-in-the-loop approaches (92–98% for crowdworkers), it falls within the confidence intervals of clinical expert performance (88.0 ± 9.00%), and the trend suggests continued improvement. Though this assumes sustained linear advancement—an assumption that warrants caution given potential diminishing returns, as evidenced in neural scaling laws and other fields of research [65–67]. Nevertheless, the current trajectory suggests that multimodal LLMs could match or perhaps even surpass the performance of humans-in-the-loop in the future, which opens up some exciting opportunities for scale and reach in diagnostics for autism (and beyond).

The narrowing performance gap reveals important patterns. When examining direct diagnosis capabilities—where models provide autism assessments without intermediate classifiers—newer models show promising results. Some earlier models even demonstrate higher direct diagnosis accuracy than their classifier-based approaches (1.5 Flash: 82.4% direct vs. 72.0% LR5), suggesting that end-to-end behavioral assessment may bypass certain limitations of feature-based classification.

The convergence of performance shown by top-performing models and human evaluators at 90% accuracy, suggests a potential performance ceiling. This performance plateau may reflect ambiguities in behavioral assessment, rather than model limitations, with both state-of-the-art LLMs and experienced human raters converging at similar performance thresholds. The consistency of this ceiling across different evaluation methods and rater types suggests that challenges in video-based behavioral assessment may require additional context or longer observation periods to overcome. While these headline performance metrics are promising, understanding the underlying reliability patterns is essential for clinical deployment.

### 5.2. Reliability and Agreement Analysis

Building on the performance findings, our reliability analysis (Figure 4) reveals nuances beneath these metrics. The “agreement paradox”—high internal consistency (*κ*_*w*_ > 0.91) coupled with moderate between-model agreement (*κ*_*w*_ = 0.636)—suggests that different architectures develop distinct assessment strategies, rather than converging on universal patterns. This pattern, visually evident in the heatmap (Figure 4) where off-diagonal LLM-to-LLM cells show moderate agreement despite each model’s internal consistency, indicates that while each model applies its learned representations consistently, different architectures develop distinct approaches to behavioral interpretation. This finding challenges assumptions about ground truth in behavioral diagnosis and raises questions about whether diagnostic consistency or diversity better serves clinical needs.

The observed agreement structure—high within-model consistency paired with moderate between-model agreement—presents both opportunities and challenges for clinical deployment. The near-deterministic behavior of individual models (*κ*_*w*_ > 0.91) ensures reproducible assessments, addressing a limitation of human evaluation where rater fatigue, training drift, and subjective interpretation introduce variability. However, the moderate between-model agreement (*κ*_*w*_ = 0.636) suggests that ensemble methods combining multiple LLMs might not yield the variance reduction benefits typically expected from aggregating independent assessments. This reliability profile contrasts sharply with human raters, who exhibit moderate agreement both within groups (*κ*_*w*_ *≈* 0.58–0.60) and between individuals. While human variability might capture genuine ambiguity in behavioral presentation, LLM consistency could either represent more reliable feature extraction or potentially indicate overfitting to specific visual patterns.

The agreement patterns also raise questions about the nature of behavioral assessment reliability. Traditional clinical practice assumes that higher inter-rater agreement indicates better assessment quality. However, our results suggest a trade-off: LLMs offer consistency at the potential cost of clinical specificity, while human raters provide varied viewpoints that may better capture the heterogeneous nature of autism presentation. Future work should investigate whether the high internal consistency of LLMs translates to improved diagnostic accuracy in prospective clinical trials, or whether maintaining some degree of assessment diversity is beneficial for capturing the full spectrum of autistic behaviors.

The systematic differences revealed in Table 2—persisting across both agreement and disagreement cases—provide evidence that models have developed stable, distinct assessment frameworks. We propose three mechanisms underlying these assessment strategies:

First, consistent strategic frameworks: The analysis of thinking outputs reveals that each model maintains its assessment approach, regardless of the final diagnosis. Gemini 2.5 Pro consistently employs clinical pattern recognition (temporal mapping, atypical behavior emphasis, exceptional skill identification), while 2.5 Flash consistently uses developmental contextualization (systematic checklists, categorical analysis, conservative interpretation). This stability within models (*κ*_*w*_ > 0.91) despite divergence between models (*κ*_*w*_ = 0.636) indicates learned strategic preferences, rather than random variation.

Second, complementary attention hierarchies: The models prioritize different behavioral channels even when observing identical footage. Pro models attend primarily to specific diagnostic markers (stimming, echolalia, hyperlexia) and behavioral patterns, while Flash models emphasize social engagement quality and developmental appropriateness. For instance, in Video V50, both concluded neurotypical, but Pro focused on whether special interests served social functions, while Flash emphasized assessment limitations.

Third, alignment with clinical schools of thought: The divergence mirrors debates in autism diagnosis. Pro’s approach aligns with DSM-5 criteria-based medical models that catalog specific deficits, while Flash’s approach reflects strength-based developmental frameworks that consider overall functioning. This parallel to human clinical training suggests that pre-training exposure to different types of behavioral content may shape these emergent assessment strategies.

The persistence of these strategies across agreement cases (Table 2, rows 4–6) demonstrates that inter-model disagreement reflects systematic philosophical differences, rather than random error. When both models diagnose autism (V15), Pro provides timestamped behavioral mapping while Flash organizes by diagnostic categories—reaching the same conclusion through entirely different analytical paths.

The feature-level reliability patterns revealed in Figure 5 have profound implications for the future development of automated autism assessment systems. The performance gradient across domains—from strong language agreement to poor stereotyped behavior detection—provides a roadmap for targeted improvements. The consistently high agreement on Expressive Language features (*κ*_*w*_ = 0.77–0.78) validates that current multimodal LLMs can reliably assess verbal communication, likely benefiting from extensive conversational data in their pre-training. However, the dramatic failure with Stereotyped Behaviors (*κ*_*w*_ = 0.08–0.30) exposes a limitation that must be addressed before these systems can achieve comprehensive diagnostic capability.

The feature-specific divergence within similar behavioral constructs particularly highlights areas requiring focused innovation. The fact that one Stereotyped Speech item achieves excellent agreement (*κ*_*w*_ = 0.774–0.782) while another shows poor concordance (*κ*_*w*_ = 0.083–0.296) suggests that subtle differences in feature operationalization significantly impact model performance. This indicates that future improvements will likely come from: (1) developing feature-specific fine-tuning approaches that target underperforming behavioral markers, (2) creating specialized attention mechanisms for detecting repetitive patterns and restricted interests that may be temporally distributed across video segments, (3) incorporating clinical knowledge graphs to better understand the relationships between different manifestations of similar behaviors, and (4) augmenting training data with synthetic examples of rare but diagnostically important behaviors.

The moderate performance on social interaction features (*κ*_*w*_ = 0.45–0.67) represents both a challenge and an opportunity. These features require integrating multiple modalities—facial expressions, body language, vocal prosody, and contextual cues—suggesting that advances in multimodal fusion architectures could lead to further improvements. The differential agreement patterns between crowdworkers and clinicians on features like Shares Excitement (*κ*_*w*_ = 0.669 vs. 0.586) further indicate that incorporating diverse human perspectives during model development could enhance robustness. As foundation models continue to evolve with better compositional understanding and temporal reasoning capabilities, we anticipate that the current performance ceiling on complex social behaviors will progressively rise, ultimately enabling comprehensive behavioral assessment comparable to expert clinical evaluation.

### 5.3. Feature Attribution and Interpretability

Complementing our agreement analysis, the difference in feature prioritization between LLMs and human raters further underscores these distinct assessment strategies. Our analysis (Figure 6 and Table 3) reveals different approaches: LLMs prioritize language-related features, with Stereotyped Speech and Expressive Language occupying three of their top five positions, while crowdworkers heavily weight social-emotional behaviors, with Shares Excitement achieving the highest importance (0.210 ± 0.090) among all features across all groups. The 3.7-fold difference in Emotion Expression importance between crowdworkers and LLMs suggests these systems may be detecting complementary rather than identical behavioral signals, thereby capturing different aspects of the autism phenotype than traditional human observation. This language-centric approach by LLMs, while achieving high accuracy, may reflect biases in their training data toward verbal communication patterns, potentially limiting effectiveness for assessing non-verbal or minimally verbal individuals who represent approximately 30% of the autism spectrum.

Feature extraction stage serves as the interpretable interface between raw behavioral observations and diagnostic decisions. Our results reveal that the success of LLM-based assessment depends on how effectively models can map video content to structured behavioral features. The divergent performance across feature categories (high for language, moderate for social, poor for stereotyped behaviors) directly traces back to the extraction process, suggesting that different behavioral domains require distinct extraction strategies. The high within-model consistency in feature extraction indicates that LLMs apply learned patterns systematically, though the moderate between-model agreement suggests multiple valid extraction approaches exist. Future improvements will likely emerge from optimizing this extraction stage through techniques such as: (1) feature-specific prompting that provides detailed behavioral anchors for each score level, (2) hierarchical extraction that first identifies broad behavioral categories before detailed scoring, (3) temporal segmentation to capture time-varying behaviors, and (4) multimodal fusion strategies that optimally combine audio and visual signals for each feature type.

### 5.4. Video Characteristics and Error Analysis

The practical implications of these assessment differences become clear when examining video-level performance (Figures 7 and 8). The effect of autism diagnosis on model-human agreement (*t*(48) = −3.704, *p* = 0.001, Cohen’s *d* = 1.048) reveals potential diagnostic bias that requires careful consideration. Videos from children with autism showed substantially higher agreement (*M* = 0.441, *SD* = 0.213) compared to neurotypical videos (*M* = 0.202, *SD* = 0.243), with this large effect size indicating that models demonstrate higher reliability when confirming autism cases than when ruling them out. This asymmetric performance could impact screening sensitivity and specificity differently across the diagnostic spectrum. This pattern implies that, while LLMs may excel in identifying clear autism cases, they may be less reliable for borderline or neurotypical cases, a limitation with significant implications for population screening, in which both false positives and false negatives are costly. The presence of negative agreement values in some videos indicates that models can actively disagree beyond chance levels, suggesting different interpretative frameworks rather than random noise. The analysis also reveals that longer videos (mean = 190.7 s for low agreement vs. 98.3 s for high agreement) paradoxically yield lower agreement, possibly because extended observations provide more opportunities for models to identify contradictory behavioral evidence. This finding challenges the assumption that more data necessarily improves diagnostic consensus and suggests that video duration and content quality require careful optimization.

Our error analysis reveals that 95% of features showed zero variance across independent runs, indicating deterministic outputs. The thinking–rating disconnect represents an architectural flaw. When Flash explicitly states “no signs of autism” yet assigns a score of 3, or when Pro declares eye contact “natural and appropriate” while missing autism entirely, they demonstrate disconnected processing pathways between verbal reasoning and diagnostic scoring. This separation means correct behavioral observation can coexist with completely contradictory diagnostic output—which is clinically unacceptable when reasoning must justify conclusions.

The combination of deterministic outputs, thinking–rating contradictions, and overconfident incorrect diagnoses indicates these models lack the calibrated uncertainty essential for clinical practice. The high variance observed in some Pro ratings versus Flash’s perfect consistency suggests different architectural approaches. Until architectures introduce appropriate stochastic variation reflecting assessment uncertainty, these systems remain unsuitable for diagnostic decisions, for which deterministic errors could have serious consequences. Beyond these inherent video characteristics, our ablation studies identify modifiable technical factors that influence performance.

### 5.5. Ablation Studies and Component Analysis

Moving from observational patterns to controlled experiments, our ablation studies (Figure 10) establish technical requirements for viable deployment. The audio requirement proves essential, with removal causing degradation (average −18.9 percentage points). The introduction of explicit reasoning capabilities through thinking mode in the 2.5 series results in average improvements of +11.5 percentage points demonstrating how structured reasoning enhances the model’s ability to integrate complex behavioral patterns. This structured reasoning likely facilitates the integration of multiple behavioral cues, temporal pattern recognition, and contextual interpretation—cognitive processes that mirror clinical assessment strategies.

The ablation study of prompt format reveals that prompt engineering must be tailored to specific model architectures, rather than assuming universal design principles. While we expected the three-choice format (including “some evidence of autism”) to reduce conservative bias and improve sensitivity, the heterogeneous responses suggest that models have developed different internal representations of diagnostic certainty, with some benefiting from graduated expression while others require binary clarity. Furthermore, the study of the context effect shows a generational progression in context independence. Earlier generations (1.5 and Flash Lite variants) show strong dependency on the structured behavioral decomposition provided via the feature-aligned questions (+16–17 percentage points with context). In contrast, the 2.5 series models have developed sufficient inherent behavioral understanding to reach accurate diagnoses through direct video analysis alone.

These components exhibit largely independent effects, rather than synergistic interactions. The performance gains from the thinking mode remain consistent regardless of the prompt format, while audio degradation uniformly impacts all configurations. This additive relationship suggests that each component addresses distinct aspects of the behavioral assessment challenge: audio provides essential diagnostic signals, thinking enables complex reasoning, context offers structured assessment for less capable models, and prompt format influences decision boundaries in model-specific ways. These findings establish guidelines for an optimal configuration: audio-visual inputs are mandatory, thinking capabilities should be activated when available, and both prompt format and context inclusion should be empirically optimized for each model generation.

### 5.6. Limitations and Future Directions

Despite the promising trajectory demonstrated in our results, there are several limitations of this study that we discuss here. The crowdworkers in our comparison dataset were selected specifically for their ability to provide answers that lead to strong performance of the LR5/10 models, rather than providing independently verified correct answers, which may create an unfair comparison when evaluating against clinicians. Additionally, our evaluation focuses on behavioral features consistent with widely used diagnostic frameworks, potentially missing novel behavioral markers that LLMs might detect but that fall outside traditional assessment approaches. The brief video segments may not capture the full complexity of behavioral presentation that emerges over longer observation periods. Furthermore, our dataset relies on parent-reported autism diagnoses from YouTube videos, rather than clinically confirmed diagnoses through standardized diagnostic instruments, which may introduce classification errors, though prior validation has shown reasonable concordance with clinical severity ratings in this dataset [17]. Additionally, prior work demonstrated that the autism cases in these 50 videos skew toward more severe presentations, creating a large separation between ASD and neurotypical groups in the behavioral feature space [17]. This severity bias likely inflates performance metrics compared to real-world screening scenarios involving subtle or early-stage presentations. Future work should evaluate models on more challenging datasets with less pronounced autism characteristics and narrower decision boundaries between diagnostic groups.

Additionally, the curated nature of the YouTube videos, with clear visibility and good recording quality, may overestimate real-world performance where videos might have poor lighting, unstable camera work, partial occlusions, or background distractions. Cultural variations in behavioral expression, parent–child interaction styles, and social norms are also not adequately represented in this primarily English-speaking, Western-context dataset. Future work should evaluate model performance on more diverse and challenging video conditions, including clinical recordings with varying quality, multicultural populations, and different home environments.

Acknowledging these constraints, this work establishes multimodal LLMs as a promising foundation for expanding access to autism detection. While current models do not quite match the best human-in-the-loop approaches, the rapid advancement observed, combined with the elimination of human infrastructure requirements, positions these systems as potentially viable alternatives to traditional assessment methods.

The robustness of these models to real-world video conditions remains an important area for future investigation. While our evaluation used curated videos with clear visibility and good quality, clinical deployment would encounter varying lighting conditions, camera angles, partial occlusions, and audio interference. Additionally, cultural factors significantly influence behavioral expression and social interaction patterns, and our primarily Western-context dataset cannot fully capture this diversity. Promisingly, the strong performance of general-purpose LLMs without task-specific training suggests that they may have learned robust representations from their diverse pre-training data that could generalize to varied conditions. Future studies should systematically evaluate performance degradation under challenging recording conditions and across culturally diverse populations to establish deployment readiness guidelines.

Translating these capabilities into accessible tools will require addressing both technical and ethical challenges. The development of lightweight model variants for resource-constrained settings, privacy-preserving federated learning approaches, and culturally adapted assessment protocols will be essential for equitable global deployment. Establishing clear regulatory frameworks adapted to foundation models, interpretability methods that translate model decisions into clinically meaningful explanations, and guidelines for accountability in AI-mediated diagnosis must precede clinical implementation. The path forward, therefore, requires careful attention to bias mitigation, clinical validation, and ethical deployment, with sustained collaboration between AI researchers, clinicians, ethicists, and affected communities to ensure that technical capabilities do not outpace deployment readiness. The technical trajectory demonstrated here suggests that automated and accurate behavioral assessments could be possible. As these models continue to improve, they offer the potential to address the challenge of measuring child developmental delays early and often in the critical windows of brain plasticity.

## 6. Conclusions

This study is an initial evaluation of the ability of multimodal large language models to measure features used by machine learning models that have validated accuracy for classifying autism versus other delays and typical development. Our analysis of seven Gemini model variants across three generations reveals performance improvements, with accuracy advancing from 72.0% in Gemini 1.5 Flash to 89.6% in Gemini 2.5 Pro using validated autism detection classifiers—a 24.4% relative improvement over 15 months of model development.

Our results highlight the potential and limitations of LLM-based behavioral assessment. While the best-performing model (Gemini 2.5 Pro) achieves 89.6% classification accuracy, approaching the 92–98% range of carefully selected crowdworkers, our analysis reveals differences in assessment strategies. LLMs demonstrate high within-model consistency (*κ*_*w*_ > 0.91) compared to moderate human agreement (*κ*_*w*_ *≈* 0.58–0.60), prioritize language-related features over the social–emotional cues that crowdworkers emphasize, and show stronger performance on videos with clear autism symptoms than those with ambiguous or complex signals. These patterns suggest that LLMs may be learning complementary behavioral signals, rather than simply replicating human judgment. Furthermore, the effect of autism diagnosis on model–human agreement (*t*(48) = −3.704, *p* = 0.001, Cohen’s *d* = 1.048) indicates asymmetric performance that must be carefully considered in clinical applications.

Our ablation studies establish technical requirements and limitations for deployment: audio input proves essential (18.9 percentage point average degradation without audio), thinking mode capabilities provide strong improvements (+11.5 percentage points on average), prompt format shows model-specific effects with binary formats generally outperforming three-choice alternatives, and newer model generations demonstrate increasing independence from structured behavioral assessment.

There are several opportunities for future work. First, extending evaluation to larger datasets, longer video segments, and diverse clinical populations, including minimally verbal individuals who represent approximately 30% of the autism spectrum, will be needed to establish generalizability. Second, developing hybrid approaches that combine LLM consistency with human clinical insight may optimize both reliability and diagnostic validity. Third, it will be useful to explore whether ensemble methods that combine multiple LLM architectures can overcome the moderate between-model agreement to achieve performance gains. Fourth, establishing regulatory frameworks and interpretability methods specific to foundation models in clinical diagnosis will be needed for responsible deployment.

The improvement trajectory observed shows promise for the use of LLMs in the process of scaling autism diagnosis. However, realizing this potential will require continued collaboration between AI researchers, clinicians, ethicists, and autism communities to ensure that technical capabilities translate into equitable, accessible, and clinically valid diagnostic tools that augment, rather than replace, human expertise.

## Figures and Tables

**Table 1.** Baseline model performance: comprehensive performance metrics across 7 Gemini models and human evaluators.

Model	Classifier	Accuracy (%)	Precision (%)	Sensitivity (%)	Specificity (%)	ROC-AUC (%)	PR-AUC (%)
2.5 Pro*	LR5	89.6 ± 2.1	89.0 ± 2.1	90.4 ± 2.7	88.8 ± 2.2	95.6 ± 0.6	96.0 ± 0.7
	LR10	87.2 ± 1.4	88.5 ± 2.0	85.6 ± 2.7	88.8 ± 2.2	91.6 ± 2.5	93.6 ± 1.9
	Direct	90.0 ± 2.5	89.7 ± 2.6	90.4 ± 2.7	89.6 ± 2.7	90.0 ± 2.5	86.2 ± 3.2
2.5 Flash *	LR5	85.6 ± 1.1	80.0 ± 1.4	96.0 ± 0.0	76.0 ± 2.2	92.6 ± 0.4	93.5 ± 0.3
	LR10	79.6 ± 1.1	76.7 ± 1.5	84.0 ± 0.0	75.2 ± 2.2	88.2 ± 0.5	88.9 ± 0.4
	Direct	85.6 ± 1.1	86.2 ± 1.9	84.0 ± 0.0	87.2 ± 2.2	86.0 ± 1.1	80.4 ± 1.6
2.5 Flash Lite Preview	LR5	63.6 ± 1.1	60.5 ± 1.0	80.0 ± 0.0	47.2 ± 2.2	66.0 ± 1.6	62.2 ± 1.3
	LR10	53.2 ± 2.2	52.2 ± 1.6	75.2 ± 2.2	30.4 ± 4.2	57.7 ± 1.1	53.5 ± 0.4
	Direct	53.6 ± 1.1	54.8 ± 1.5	39.2 ± 2.2	68.0 ± 0.0	54.0 ± 1.1	52.2 ± 0.6
2.0 Flash *	LR5	80.0 ± 0.0	75.9 ± 0.0	88.0 ± 0.0	72.0 ± 0.0	88.1 ± 1.1	89.7 ± 1.1
	LR10	72.0 ± 0.0	66.7 ± 0.0	88.0 ± 0.0	56.0 ± 0.0	84.6 ± 0.7	85.2 ± 0.6
	Direct	78.0 ± 0.0	73.3 ± 0.0	88.0 ± 0.0	68.0 ± 0.0	78.0 ± 0.0	70.5 ± 0.0
2.0 Flash Lite	LR5	69.5 ± 2.5	64.6 ± 1.9	89.2 ± 2.7	49.7 ± 2.2	83.0 ± 1.9	75.8 ± 1.6
	LR10	65.5 ± 1.8	59.6 ± 1.6	95.6 ± 0.0	34.7 ± 3.2	85.2 ± 0.7	80.5 ± 1.6
	Direct	82.7 ± 3.3	80.8 ± 4.8	86.4 ± 2.7	79.1 ± 6.3	82.7 ± 3.3	76.3 ± 4.2
1.5 Pro	LR5	80.0 ± 0.0	94.1 ± 0.0	64.0 ± 0.0	96.0 ± 0.0	91.8 ± 0.3	89.0 ± 1.1
	LR10	76.0 ± 2.5	93.3 ± 0.6	56.8 ± 5.0	96.0 ± 0.0	87.6 ± 0.5	85.4 ± 1.0
	Direct	72.4 ± 2.1	92.5 ± 0.6	48.8 ± 4.2	96.0 ± 0.0	72.4 ± 2.1	70.6 ± 2.1
1.5 Flash	LR5	72.0 ± 1.8	85.4 ± 5.7	52.8 ± 2.2	91.2 ± 4.2	81.3 ± 0.9	82.6 ± 1.0
	LR10	78.8 ± 2.2	78.1 ± 2.3	80.8 ± 3.5	76.8 ± 2.7	83.6 ± 1.2	63.6 ± 1.6
	Direct	82.4 ± 1.1	76.3 ± 1.4	94.4 ± 2.7	70.4 ± 2.7	82.4 ± 1.1	75.0 ± 1.2
Clinicians	LR5	88.0 ± 9.0	100.0 ± 0.0	76.0 ± 16.9	100.0 ± 0.0	98.1 ± 2.9	98.4 ± 2.6
	LR10	98.0 ± 3.0	100.0 ± 0.0	96.0 ± 6.8	100.0 ± 0.0	99.0 ± 1.8	99.2 ± 1.6
Crowdworkers [17]	LR5	92.0–98.0 ± 3.0–7.0	92.0–100.0 ± 0.0–10.4	88.0–96.0 ± 6.8–13.0	92.0–100.0 ± 0.0–10.4	99.0–99.4	99.1–99.4
	LR10	90.0–96.0 ± 5.0–8.0	85.7–100.0 ± 0.0–12.4	92.0–96.0 ± 6.8–10.0	84.0–100.0 ± 0.0–13.7	98.5–98.7	98.9–99.0

* indicates best-performing models.

Note. AI model results are reported as mean ± 95% confidence intervals across five independent runs (t-interval); clinician and crowdworker results are mean ± 95% confidence intervals computed using bootstrap resampling (crowdworker data from [17]). Baseline: Video+Audio input, binary diagnosis prompt; “thinking” enabled for 2.5 series; the diagnosis question presented alongside the other behavioral questions (context). LR5/LR10 denotes logistic regression with 5/10 features. Direct denotes the direct diagnosis approach (unavailable for human evaluators). Clinician scores use mean aggregation; crowdworker scores show ranges over mean/mode/median aggregation from [17].

**Table 2.** Divergent assessment strategies between Gemini models: Different assessment strategies persist regardless of diagnostic agreement. Claude Opus 4.1 was used to separate the reasoning outputs into the themes below.

Video	Ground Truth	Agree?	Gemini 2.5 Pro Strategy	Gemini 2.5 Flash Strategy
Cases with Different Diagnoses (Disagreement)				
V1	ASD	No	Diagnosed: ASD Atypical behavior emphasis: “Name called, no response. That’s a red flag”; “arm flapping is a classic stim”; “lining them up… restrictive, repetitive”	Diagnosed: NT Strength emphasis: “Clear positive social interaction”; “joint attention and shared activity”; “typical developmental trajectories”
V20	NT	No	Diagnosed: NT Social competency focus: “Initiating with joke… understands social nuances, humor”; “excellent social-emotional reciprocity”	Diagnosed: ASD Pattern recognition: “Repetitive question eliciting repetitive response”; “could fall under echolalia”; “lacks genuine turn-taking”
V69	ASD	No	Diagnosed: ASD Exceptional skill flagging: “2.5-year-old knowing capitals is highly abnormal”; “example of hyperlexia… points to ASD”	Diagnosed: NT Social priority: “Child seems quite engaged”; “maintaining eye contact”; “developmental trajectory appears typical”
Cases with Same Diagnoses but Different Strategies (Agreement)				
V3	NT	Yes	Diagnosed: NT Performance assessment: “Not just singing; he is performing”; “showing social awareness and engagement”; “obvious give and take”	Diagnosed: NT Systematic checklist: “Run through typical indicators for ASD”; “could be motor mannerisms… also common for neurotypical children”
V15	ASD	Yes	Diagnosed: ASD Temporal mapping: “0:38–0:42: Caregiver calls name… glances briefly”; “1:15–1:20: Repetitive hand movements”; chronological pattern analysis	Diagnosed: ASD Categorical analysis: “1. Social Interaction Deficits”; “2. Communication Challenges”; “3. Restricted, Repetitive Patterns”
V50	NT	Yes	Diagnosed: NT Cognitive contextualization: “Interest in capitals is intense, but… it’s the way she engages with the adult”; evaluates social function of interests	Diagnosed: NT Conservative assessment: “Video is extremely short… lacks context”; “impossible to make reliable assessment”; defaults to NT when uncertain

ASD = Autism Spectrum Disorder; NT = Neurotypical.

**Table 3.** Top-5 features per group using permutation and random forest importance rankings. Consensus rank is computed as the mean of the two method ranks; ties are broken using the better random forest rank.

Group	Consensus Rank	Feature	Permutation	Random Forest
LLMs				
	1	Stereotyped Speech-1	2	2
	2	Expressive Language-2	4	1
	3	Expressive Language-1	3	3
	4	Eye Contact	1	5
	5	Shares Excitement	5	4
Crowdworkers				
	1	Shares Excitement	3	1
	2	Eye Contact	1	4
	3	Emotion Expression	4	2
	4	Social Overtures	5	3
	5	Communicative Engagement	2	8
Clinicians				
	1	Expressive Language-2	3	1
	2	Expressive Language-1	2	2
	3	Stereotyped Speech-1	1	3
	4	Speech Patterns-2	6	4
	5	Eye Contact	7	5

Note. Numerical suffixes (−1, −2) distinguish features adapted for different developmental levels.

## Data Availability

The data presented in this study are available upon request from the corresponding author due to Stanford University’s data use restrictions.

## Appendix A. Gemini Model Prompt and Behavioral Assessment Questions

### Appendix A.1. Standardized Prompt for Gemini Models

The following prompt was provided to all Gemini model variants:

You are a behavioral analyst observing short videos of children interacting with a caregiver. Your task is to answer a set of structured multiple-choice questions about the child’s behavior based on the video. Each question is scored on a scale from 0 to 3. You should provide the most appropriate score based on your observation. Even if the behavior is not directly shown, use indirect evidence (e.g., body language, facial expression, context) to infer the most likely behavior.

### Appendix A.2. Direct Diagnosis Prompt Variations

For the direct diagnosis approach and prompt format ablation studies, we tested two variations of the final assessment question:

#### Appendix A.2.1. Binary Format

**Based on the video evidence, does this child show characteristics consistent with autism?**

- (0) No, the child’s behavior appears neurotypical
- (1) Yes, the child shows behavioral patterns consistent with autism

#### Appendix A.2.2. Three-Choice Format

**Based on the observed behaviors, evaluate the likelihood of autism:**

- (0) No evidence of autism
- (1) Some evidence of autism
- (2) Strong evidence of autism

Note: For the three-choice format, responses of (1) “Some evidence” and (2) “Strong evidence” were both coded as positive for autism diagnosis in the binary classification task.
